# Isolation and Molecular Characterization of Two Novel Lytic Bacteriophages for the Biocontrol of *Escherichia coli* in Uterine Infections: In Vitro and Ex Vivo Preliminary Studies in Veterinary Medicine

**DOI:** 10.3390/pharmaceutics14112344

**Published:** 2022-10-30

**Authors:** Victor M. Balcão, Bianca G. Belline, Erica C. Silva, Pablo F. F. B. Almeida, Denicezar Â. Baldo, Lara R. P. Amorim, José M. Oliveira Júnior, Marta M. D. C. Vila, Fernando S. Del Fiol

**Affiliations:** 1 PhageLab, Laboratory of Biofilms and Bacteriophages, University of Sorocaba, Sorocaba 18023-000, Brazil; 2 Department of Biology and CESAM, Campus Universitário de Santiago, University of Aveiro, P-3810-193 Aveiro, Portugal; 3 Department of Education, Faculty of Sciences, University of Porto, P-4169-007 Porto, Portugal

**Keywords:** bacteriophage, *Myoviridae* genus *Asteriusvirus*, *Myoviridae* genus *Tequatrovirus*, *E. coli*, antibacterial phage activity, pyometra, phage stabilization and delivery

## Abstract

*E. coli* is one of the etiological agents responsible for pyometra in female dogs, with conventional treatment involving ovariohysterectomy. Here, we report the isolation and full characterization of two novel lytic phages, viz. vB_EcoM_Uniso11 (ph0011) and vB_EcoM_Uniso21 (ph0021). Both phages belong to the order Caudovirales and present myovirus-like morphotypes, with phage ph0011 being classified as *Myoviridae* genus *Asteriusvirus* and phage ph0021 being classified as *Myoviridae* genus *Tequatrovirus*, based on their complete genome sequences. The 348,288 bp phage ph0011 and 165,222 bp phage ph0021 genomes do not encode toxins, integrases or antimicrobial resistance genes neither depolymerases related sequences. Both phages were shown to be effective against at least twelve *E. coli* clinical isolates in in vitro antibacterial activity assays. Based on their features, both phages have potential for controlling pyometra infections caused by *E. coli.* Phage ph0011 (reduction of 4.24 log CFU/mL) was more effective than phage ph0021 (reduction of 1.90 log CFU/mL) after 12 h of incubation at MOI 1000. As a cocktail, the two phages were highly effective in reducing the bacterial load (reduction of 5.57 log CFU/mL) at MOI 100, after 12 h of treatment. Both phages were structurally and functionally stabilized in vaginal egg formulations.

## 1. Introduction

The incorrect prescription and indiscriminate use of antibiotics have determined bacterial selection and resistance, resulting in infections that are increasingly difficult to treat, implying high-cost therapies and greater toxicity for the patient, being directly related to the emergence of the so-called “super-bacteria”, which are bacterial strains resistant to virtually all antibiotics currently available in the antibacterial arsenal [1]. Veterinary Medicine has its share of responsibility in this matter, mainly in the sphere of animal production with the use of antimicrobials in sub-therapeutic doses as growth promoters, making animals reservoirs of resistance genes capable of transmission to humans [2,3,4]. The bacteria *E. coli* is one of the most prevalent microorganisms in bacterial infections in Veterinary Medicine, being frequently associated with infections of the reproductive tract, including the mammary glands, and urinary tract infections [5,6,7]. Although naturally present in the intestinal tract of animals, in the face of imbalances in the bacteria-host relationship, diseases appear responsible for enormous economic losses in animal production [8]. Bacterial infections by *E. coli* are largely related to reduced reproductive efficiency in different species, such as bovine mastitis and metritis [9,10,11,12,13], endometritis in mares [14,15,16,17] and pyometra (uterine infections) in female dogs and cats [18]. In the case of canine and feline pyometra, the bacterial infection is secondary to hormonal changes in estrus after estrus, with ascending contamination by bacteria usually originating from the vagina [19,20,21]. The literature demonstrates the prevalence of *E. coli* isolates in the analysis of uterine contents collected after pyometra [22,23,24,25,26,27,28], evidencing characteristics of bacterial resistance to the main antibiotics used [22,24,27]. The absence of an effective response to antibiotic therapy makes the treatment of choice for canine pyometra to be ovariohysterectomy (OH), compromising the female dogs reproductive life and resulting in economic losses to commercial kennels [29]. Pyometra is a disease of relevant importance in Veterinary Medicine due to the high incidence and systemic involvement of females, which can rapidly progress to sepsis and death, hence early diagnosis and intervention are essential [30]. Bacterial resistance to antibiotics is a worldwide concern, so the research and feasibility of alternative treatments are extremely necessary, especially given the high cost and difficulties inherent in obtaining new antibiotics [31]. Among the alternatives to conventional antibiotics, phage therapy is emerging as an effective and safe option for the treatment of bacterial infections [32,33,34,35,36,37,38].

Although there are already published studies carried out in vitro, such as the one by Guo et al. [39], which isolated and characterized lytic phages for *E. coli* aiming at therapeutic application in bovine mastitis, data regarding the effectiveness and safety of in vivo applications are scarce, hence it is important to expand research regarding application routes, pharmacokinetics and pharmacodynamics of phages in phage therapy. When bacteriophages infect their bacterial host cell, they take control of the host’s metabolic machinery and thus become able to interact, replicate and evolve, with such interactions being complex and not entirely predictable [35,36]. Although phage therapy presents itself as a viable alternative with promising results [35,36], more research is needed on its pharmacokinetics, including studies on the ability of phage particles to self-replicate, biological and genomic characterization, possible adverse effects related to the immune system, possible interactions with the eukaryotic cells of the animal host, and delivery systems [40,41,42,43,44,45]. Among the advantages of phage therapy, the high specificity of phage-bacteria stands out [34,35], not requiring successive doses of the bacteriophage, since replication occurs in the host bacterium as long as viable bacterial cells subsist, and ceases when host bacterium cells are no longer present [35,36,46]. Additionally, when using phage therapy, secondary or opportunistic infections such as those caused by empirical antibiotic therapy are also avoided, which affect both pathogens and part of the patient’s natural microbiota, often resulting in dysbiosis and bacterial translocation [34]. Hence, phages reappear in the current scenario of high bacterial resistance to conventional antibiotics as potential alternatives to these antimicrobials [34,35,36,46]. Although it is possible for bacteria to develop resistance to phages by several mechanisms, including conformational changes in surface receptors, studies already carried out show that the use of a cocktail of phage particles increases the spectrum of action, reducing the appearance of resistant bacterial phenotypes. Moreover, the isolation of new lytic phages for a given bacterial strain is much simpler and more economically viable than the development of new antibiotics [34,35,36]. Phages with the potential for therapeutic use need to be extensively characterized both from a biological and genomic point of view [36], before any clinical trial and use in antibacterial therapy, since only strictly lytic phages, also called virulent, must be selected [34,35,36,46]. As for possible side effects resulting from the use of phages to fight bacterial infections, the release of endotoxins due to bacterial lysis has been described in the literature, but this also occurs in conventional antibiotic therapy [1].

In the research work described herein, we report the isolation and full characterization of two novel lytic phages against *E. coli*, aiming at potential treatments of animal uterine infections by this bacterium (pyometra). Moreover, the efficiency of the two novel phages against *E. coli* strains from clinical isolates was also investigated, together with in vitro and ex vivo *E. coli* inactivation trials. Finally, a vaginal egg-type delivery system for a cocktail integrating both lytic phages was developed and fully characterized.

## 2. Materials and Methods

### 2.1. Biological Material

The bacterial strain of *E. coli* (CEFAR CCCD-E003), utilized in this study as the phage host, was acquired from CEFAR Diagnóstica (São Paulo/SP, Brazil). Two phages were isolated from samples of sewage water, collected on 9 December 2021 from the Veterinary Hospital at UNISO (Sorocaba/SP, Brazil). The biological samples from which the analyzed bacterial clinical isolates were obtained were kindly provided by the Veterinary Hospital of the University of Sorocaba (Sorocaba/SP). Biological materials were collected from uteri of female dogs removed in ovariohysterectomy procedures, using sterile swabs, and placed in a Cary Blair™ (Sigma-Aldrich, St. Louis, MO, USA) type transport medium. *E. coli* CCCD-E003, *P. mirabilis* CCCD-P001, *E. faecalis* CCCD-E002, *S. epidermidis* CCCD-S010, *S. enterica* CCCD-S004, *P. aeruginosa* CCCD-P004, *B. subtilis* CCCD-B010, *S. aureus* CCCD-S009 and *K. pneumoniae* CCCD-K001 were acquired from CEFAR (São Paulo SP, Brazil). *E. coli* (ATCC 25922) was acquired from ATCC—American Type Culture Collection (Gaithersburg, Maryland, MD, USA).

Cultures of all bacteria were maintained in solid TSA (Tryptic Soy Agar, Sigma-Aldrich Brazil (Cotia/SP, Brazil)) at 4 °C and, previously to each assay, one single colony was transferred to 25 mL of liquid TSB (Tryptic Soy Broth, also from Sigma-Aldrich Brazil (Cotia/SP, Brazil)) and incubated overnight at 37 °C. From this culture, 100 µL was transferred into 10 mL of recently prepared TSB and the resulting suspension was incubated overnight at 37 °C so as to attain an optical density at 600 nm of 1.0 (OD_600nm_ = ca. 1.0, corresponds to ca. 10^9^ cells per mL).

Cell lineage HaCaT (in vitro spontaneously transformed keratinocytes from histologically normal human skin) was acquired from CLS Cell Lines Service GmbH (Eppelheim, Germany) whereas cell lineage V79 (chinese hamster lung fibroblasts, ATCC CCL-93) was acquired from Banco de Células do Rio de Janeiro (Rio de Janeiro RJ, Brazil). The cells were kept at 37 °C in a moist atmosphere containing 5% CO_2_, in Dulbecco’s Modified Eagle’s Medium (DMEM).

### 2.2. Chemicals

All reagents used were of analytical grade or better, and were used without further purification. Anhydrous Na_2_HPO_4_, NaH_2_PO_4_, CaCl_2_ and NaCl were purchased from Dinâmica Química Contemporânea Ltd.a (Diadema/SP, Brazil). MgSO_4_ was supplied by Sigma-Aldrich (St. Louis, MO, USA). TSA and TSB were purchased from Sigma-Aldrich Brazil (Cotia/SP, Brazil). Bacteriologic agar was from Gibco Diagnostics (Madison, WI, USA). Polyethylene glycol (PEG) 8000 was from Sigma-Aldrich (St. Louis, MO, USA). “Enterokit B” was acquired from PROBAC DO BRASIL Produtos Bacteriológicas Ltd.a. (São Paulo/SP, Brazil), and consisted of the following culture media: EPM, MILi and Simmons Citrate, in a box with 16 sets and a vial of Kovacs reagent. The Stericup™—GP sterilizing filtration systems (0.22 µm pore diameter polyethersulphate membrane) were acquired from Merck-Millipore (Darmstadt, Germany). Deodorized cocoa butter (lot. 04DS02) was purchased from BrazilCoa Com. de Prod. Ltd.a. (Indaiatuba/SP, Brazil). Ultrapure water was produced in a Master System All MS2000 (Gehaka, São Paulo/SP, Brazil) to a final resistivity of 0.1818 MΩ·m and conductivity of 5 µS·m^−1^.

### 2.3. Isolation and Characterization of the Bacterial Strains Responsible for Animal Uterine Infections (Pyometra)

The biological material was collected from uteri surgically removed in ovariohysterectomy procedures. The uterine surface was cleaned with antiseptic (3% chlorhexidine, *v*/*v*), incised with sterile blades and rubbed with sterile swabs. Swabs were transported in Cary Blair™ type medium and plated afterwards in test tubes containing TSB liquid medium for overnight enrichment at 37 °C and then streaked into Petri plates containing TSA solid culture medium, which were also incubated overnight at 37 °C for colony growth and isolation. After incubation, an isolated colony (CFU) was aseptically removed using a sterile loop and added to a glass slide with a drop of saline solution, followed by Gram staining. It was also plated on TSA culture media and on the selective media MacConkey™ agar (Petri plates were incubated overnight at 37 °C) to identify the bacteria. In addition to observing the growth and morphology of colonies, a lactose fermentation test was performed, as well as specific biochemical tests to identify and characterize the isolated bacterial species. The differentiation of bacterial genus and species was made through a series of biochemical tests, aiming to investigate the activities inherent to bacterial metabolism, since each microorganism has its own specific enzymatic system. Such tests require special culture media with the substrate to be analyzed, indicating its appropriate use by the bacteria under scrutiny. Enterokit B (PROBAC do Brasil—Produtos Bacteriológicos Ltd, São Paulo/SP, Brazil) was used in this research work, and contained the following media: EPM, MlLi and Simmons Citrate. The EPM medium integrated the fermentation and gas production tests on glucose, H_2_S production, urea hydrolysis and tryptophan deamination. MlLi medium integrated motility, indole and lysine decarboxylation tests. Simmons Citrate Medium provided citrate as the sole carbon source. The three media contained eight biochemical tests which, together with fermentation of lactose in the isolation plate, allowed for the reliable identification of the vast majority of Enterobacteriaceae isolated from clinical samples of the uterus of ovariohysterectomized female dogs.

### 2.4. Phage Enrichment, Isolation and Enumeration

Phage enrichment from hospital wastewater was performed according to Balcão et al. [46] and Harada et al. [36]. Wastewater samples (50 mL) were collected from Veterinary Hospital at UNISO (Sorocaba/SP, Brazil) on 9 December 2021, mixed with an overnight culture of *E. coli* (CEFAR CCCD-E003) (in exponential growth) (50 μL) and added with TSB (50 mL), incubated at 37 °C for 24–48 h, centrifuged at 9000× *g* and 4 °C during 10 min. The supernatant was then collected and filtered through Stericup™—GP filtration system (Merck-Millipore, Darmstadt, Germany). To verify if phages were present in the enriched supernatant, spot-testing of 10 µL-droplets of the filtered supernatant was performed on a lawn of *E. coli* CCCD-E003.

Isolation of phage plaques was carried out using the conventional double-layer agar method described in detail elsewhere [36,46,47,48]. The phage suspensions were centrifuged (9000× *g*, 4 °C, 10 min) to remove intact bacterial cells or bacterial debris, and this procedure was repeated until all phage plaques exhibited the same morphology.

Titers of phage suspensions (PFU/mL, plaque forming units/mL) were then determined according to the procedure described elsewhere [36].

### 2.5. Phage PEG-Precipitation

The phage suspensions (with titers ≥10^10^ PFU/mL) were added with a mixture of sterile PEG 8000 (10%, *w*/*w*) containing 1 M NaCl, in a volumetric proportion of 2:1, respectively. The resulting mixture was incubated overnight at 4 °C and centrifuged at 11,000 rpm and 4 °C during 45 min. Afterwards, the supernatant was carefully discarded and the pellet resuspended and homogenized in 5 mM MgSO_4_.

### 2.6. UV-Vis Spectral Scans for Determination of Phage Particle Molar Extinction Coefficient

The spectrophotometric assay carried out was based on the procedure described elsewhere [36,46,47,49].

### 2.7. SDS-PAGE Analysis of Phage Structural Proteins

The structural protein profile of the purified phage particles was studied by sodium dodecyl sulphate-polyacrylamide gel electrophoresis (SDS-PAGE), following the procedure described elsewhere [36,46,47], using 1.2 × 10^11^ virions of phage vB_EcoM_Uniso11 and 1.1 × 10^11^ virions of phage vB_EcoM_Uniso21.

### 2.8. Transmission Electron Microscopy (TEM) Analyses

Phage particles of PEG-concentrated suspensions were negatively stained with uranyl acetate (Sigma-Aldrich, St. Louis, MO, USA) at 2% (*w*/*v*) and pH 7.0, following the procedure described elsewhere [46]. The electron microscopy analyses were performed in a Transmission Electron Microscope from JEOL (model JEM 2100, Tokyo, Japan), encompassing a LaB_6_ filament, operating at 200 kV and with resolution of 0.23 nm; a high-resolution CCD camera from GATAN Inc. (model ORIUS™ 832.J4850 SC1000B, Pleasanton, CA, USA) with a resolution of 11 Mp (4.0 × 2.7 k pixels/9 × 9 µm^2^) was utilized for the acquisition of digital images, via software Gatan Microscopy Suite (DigitalMicrograph from Gatan Inc., version 2.11.1404.0).

### 2.9. Determination of Phage Particles Physical Parameters

The PEG-concentrated phage suspensions (diluted to 33.3% (*v*/*v*) with SM buffer) and the *E. coli* (CEFAR CCCD-E003) (at dilutions 1:10 and 1:100) were analyzed by Dynamic Laser Light Scattering (DLS) following the procedure described elsewhere [36].

### 2.10. Host Range of Isolated Phage Particles: Spot Test and Efficiency of Plating (EOP)

Phage host-range was evaluated via spot testing using the bacterial strains displayed in Table 1, following the procedure described elsewhere [36,46,50,51]. In addition to the isolation strain *E. coli* CCCD-E003, the other collection strains used to assess the host range were *S. enterica* CCCD-S004, *P. aeruginosa* CCCD-P004, *P. mirabilis* CCCD-P001, *E. faecalis* CCCD-E002, *B. subtilis* CCCD-B010, *S. epidermidis* CCCD-S010, *S. aureus* CCCD-S009, *K. pneumoniae* CCCD-K001 and *A. baumannii* ATCC-19606. Bacterial strains obtained from the clinical isolates (9 isolated strains of *E. coli*) and bacterial strains originating from other ongoing research projects at PhageLab-UNISO and Labiton-UNISO were also tested, viz. *Enterobacter* sp. (2.2; ref. Labiton), *Enterobacter aerogenes* (2.13; ref. Labiton), *E. coli* (3.2, 3.4, 3.5; ref. Labiton), *K. pneumoniae* (4.15; ref. Labiton), *Proteus penneri* (5.5; ref. Labiton), *P. vulgaris* (5.4, ref. Labiton), *S. intermedius* (ref. PhageLab), *Pseudomonas syringae* pv. *syringae* (ref. DSM 21482, from Leibniz-Institute DSMZ—Deutsche Sammlung von Mikroorganismen und Zellkulturen GmmH (Braunschweig, Germany)), *Pseudomonas syringae* pv. *garçae* (ref. IBSBF-158, from Phytobacteria Culture Collection of Instituto Biológico (IBSBF, Campinas/SP, Brazil)), and *Xanthomonas axonopodis* pv. *citri* 306 (isolated from *Citrus sinensis* at Paranavaí/PR, 1997, and kindly gifted by USP (São Paulo, Brazil)). Sensitivity of the different bacteria to the phages was deduced from formation of a clear lysis zone at the spot, differentiating the bacteria depending on the translucency produced at the spotted zone as either positive (clear lysis zone) or negative (no-lysis zone). For those bacteria with positive spot tests, the EOP was determined via the double-layer agar method [48]. The EOP for each bacterial strain was calculated considering the EOP value of 100% for *E. coli* CCCD-E003 (strain used for phage isolation), as $\mathrm{EOP}=\{\left({\mathrm{Average}\;\mathrm{PFU}}_{\mathrm{target}\;\mathrm{bacteria}}\right)/\left({\mathrm{Average}\;\mathrm{PFU}}_{\mathrm{host}\;\mathrm{bacteria}}\right)\}\times 100$ [36,46,50,52]. The values displayed in Table 1 for the EOP are the average of three independent determinations, and were scored [53] following the procedure described elsewhere [46].

### 2.11. One-Step Growth Curves (OSGC)

The growth parameters for phages ph0011 and ph0021 were obtained from the OSGC using *E. coli* CCCD-E003 and ph0011 and ph0021 at MOI ≤ 0.001, as described elsewhere [36,46], with three independent experiments performed.

The experimental data was then nonlinear-fitted to a typical sigmoidal curve (4-parameter logistic regression (4-PL) model), viz. $\mathrm{Log}\;\left(P_{t}\right)={\;P}_{\infty }+\left(\left\{P_{0}-P_{\infty }\right\}/\left\{1+{\left(t/A\right)}^{B}\right\}\right)$, so as to allow determination of the phage growth parameters (eclipse, latent and intracellular accumulation periods, and burst size) [36,50], with P_t_ representing the phage concentration (PFU/mL) at time t, P_0_ representing the phage concentration (PFU/mL) at time zero, P_∞_ representing the phage concentration (PFU/mL) at t = ∞, A representing the point of inflection of the OSGC curve, B representing the Hill’s slope that define the steepness of the OSGC curve, and t representing the time (min). The Solver function of Microsoft Excel (Microsoft, Redmond, WA, USA) was used to perform the nonlinear fitting of the phage growth data to the 4-PL model.

### 2.12. Adsorption Rate

Phage adsorption rate was determined as described elsewhere [36,46]. A bacterial suspension of *E. coli* CCCD-E003 was prepared in 25 mL of TSB and incubated at 37 °C until exponential growth. Optical density (OD_610nm_ ≈ 0.5) of the bacterial suspension to be infected by the bacteriophage particles was used to obtain a MOI of 0.001 [54], thus ensuring that there was a bacterial cell for a phage particle to adsorb onto. For this, a dilution of the phage suspension was prepared until a concentration of 8 × 10^5^ PFU/mL was obtained, which was then added to 2 mL of the bacterial suspension in a 15 mL Falcon tube. The resulting suspension was gently homogenized with a micropipette and, after 30 s, an aliquot of 50 μL was withdrawn (time 0) and serial dilutions were performed up to 10^−5^. Aliquots of the mixture were then collected at 5 min intervals until 30 min, followed by sampling at 10 min intervals until 150 min of assay was completed. The 50 μL aliquots collected were added to 425 μL of TSB liquid medium containing 25 μL of chloroform, aiming to inactivate bacterial cells at the 10^−1^ dilutions. In the following dilutions, from 10^−2^ to 10^−5^, no chloroform was added. Three droplets of 5 µL of each dilution were then plated in Petri plates previously marked with the corresponding dilutions, already prepared with a bacterial lawn of *E. coli* CCCD-E003 (produced with 100 µL of bacterial suspension and 5 mL of MTA-TSB) on solidified TSA, and the inoculum was allowed to dry. The Petri plates were then incubated inverted overnight at 37 °C, and examined for the presence of lysis plaques. Three independent assays were performed. Phage adsorption was expressed as the decrease in phage titer in the supernatant (%) compared to time zero, i.e., normalized phage concentration was followed during the experiment timeframe.

Hypothesizing that the phage particles in suspension together with the planktonic bacterial cells are able to adsorb onto susceptible cells and form a reversible complex integrating phage and bacteria that may, or not, produce an infected bacterium, viz. $\mathrm{Free}\;\mathrm{phage}\;\left(P\right)+\mathrm{bacteria}\;\left(X_{0}\right)\;\begin{array}{c} \delta \cdot X_{0}\\ ⇄\\ \phi \end{array}\;\mathrm{Reversible}\;\left\{\mathrm{phage}-\mathrm{bacteria}\right\}\;\mathrm{complex}\;\left(\Delta \right)$, the mathematical model $\frac{P_{t}}{P_{0}}=\frac{\phi }{\delta \cdot X_{0}+\phi }\left\{1+\frac{\delta \cdot X_{0}}{\phi }\cdot e^{-\phi \left(\frac{\delta \cdot X_{0}+\phi }{\phi }\right)\cdot t}\right\}$ [55,56] was nonlinear fitted to the experimental phage adsorption data, allowing to estimate the phage particles adsorption rate onto the bacterial host cells. In the mathematical model just described, P_t_ is phage concentration (PFU/mL) at time t, P_0_ is phage concentration (PFU/mL) at time zero, δ is the (first order) phage adsorption rate onto susceptible bacterial cells (PFU^−1^ CFU^−1^ mL^−1^ min^−1^), ϕ is the (first order) phage desorption rate from reversible phage-bacteria complexes (mL min^−1^), X_0_ is the initial concentration of uninfected (but susceptible) bacterial cells (CFU/mL) and t is the infection time (h). The Solver function of Microsoft Excel (Microsoft, Redmond, WA, USA) was used to perform the nonlinear fitting of the phage adsorption data to the mathematical model just described.

### 2.13. In Vitro Phage-Bacteria Inactivation Assays

Inactivation of planktonic *E. coli* CCCD-E003 bacterial cells (10^5^ CFU/mL, final concentration, in the exponential growth phase) by the two phage particles was studied at MOI values of 0.1 (10^4^ PFU/mL), 1 (10^5^ PFU/mL), 100 (10^7^ PFU/mL) and 1000 (10^8^ PFU/mL). For each phage-bacteria inactivation assay, a bacterial control (BC) and a phage control (PC) were also included. The BC was inoculated with planktonic bacterial *E. coli* CCCD-E003 cells only and the PC was inoculated with phage only. Controls and test samples were incubated under the same conditions. Aliquots of test samples (BP, bacteria plus phage) and of BC and PC were withdrawn at times 0, 2, 4, 6, 8, 10, 12 and 24 h of incubation. The phage titer in the aliquots was determined in triplicate via the double agar-layer method [48] using 5 µL droplets plated in triplicate on a bacterial lawn (exponential growth phase, OD_610nm_ ≈ 0.5), after 12 h of incubation at 37 °C, whereas the bacterial concentration in the aliquots was determined in triplicate in solid TSA medium by plating 5 µL-droplets, after 12 h of incubation at 37 °C. Three independent assays were performed.

### 2.14. Ex Vivo Phage Treatment Experiments in Artificially Contaminated Uterus

**Figure 1:**
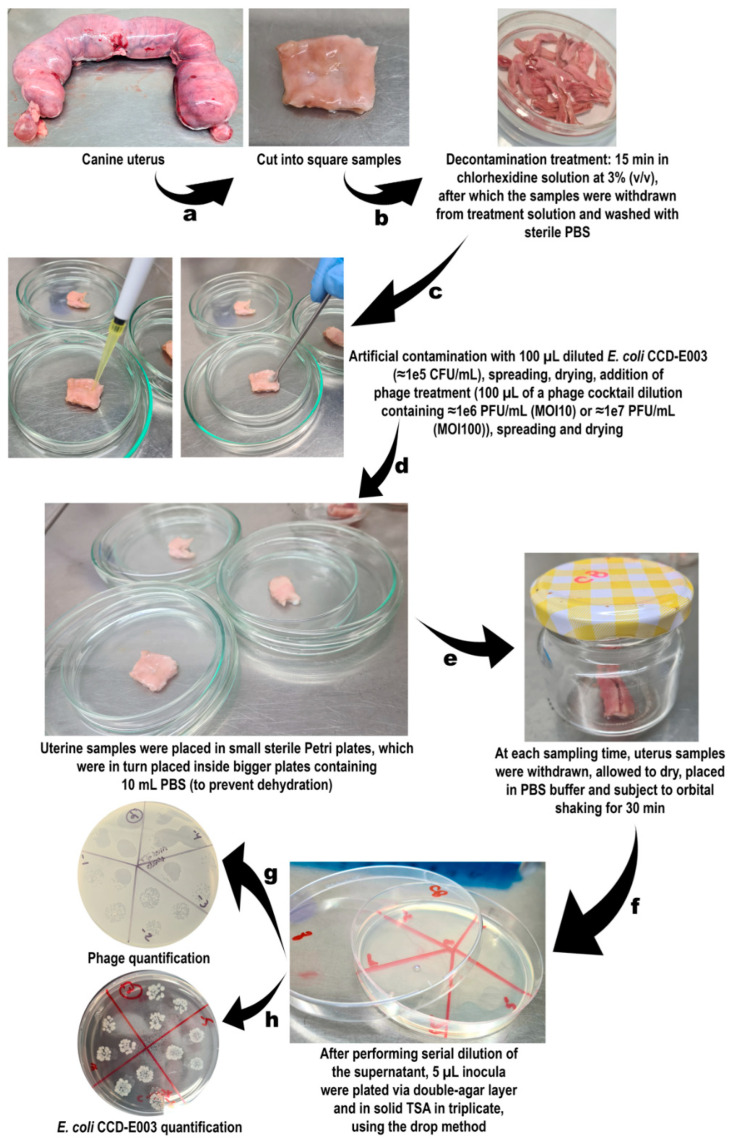
Simplified scheme of the ex vivo phage treatment procedure using the cocktail with phages vB_EcoM_Uniso11 and vB_EcoM_Uniso21 on artificially contaminated canine uterus. Legend: (**a**) canine uteruses were cut into square (ca. 2 cm × 2 cm) samples; (**b**) square samples of canine uterus were subject to decontamination by immersion in an aqueous solution of chlorhexidine at 3% (*v*/*v*) during 15 min and, following decontamination, the samples were washed with sterile PBS buffer; (**c**) after being fully decontaminated, the uterus samples were artificially contaminated with *E. coli* suspension and allowed to dry, after which they were added with the phage cocktail suspension (at MOI of either 10 or 100) and equally allowed to dry; (**d**) during the phage treatment, all uterus samples were maintained in a moist environment to prevent dehydration; (**e**) at pre-determined intervals of time, uterus samples were withdrawn from the moist environment and submerged in PBS buffer under orbital shaking so as to allow transference of viable *E. coli* cells and phage particles into the supernatant; (**f**) the supernatant was ten-fold diluted for both *E. coli* cells and phage particles quantification; (**g**) phage concentration was determined via the double-layer agar method after an incubation of 12 h at 37 °C; (**h**) bacterial concentration was determined in triplicate by the drop-plate method in TSA after an incubation of 12 h at 37 °C.

Canine uteri were kindly provided by the Veterinary Hospital of the University of Sorocaba (Sorocaba/SP, Brazil), originating from female dogs subjected to ovariohysterectomy procedures, and were kept frozen at −20 °C. Before performing the phage-bacteria inactivation assays, all uteri samples were labelled, cut into small squares (2 cm × 2 cm) and duly sterilized via immersion in chlorhexidine solution at 3% (*v*/*v*) for 15 min. Twelve groups of uteri samples (4 groups for each incubation time) were used in these assays. Each group included 3 replicates of a uterus sample (total of 12 groups × 3 replicates = 36 square uterus samples). Each replicate was placed in an independent (sterile) Petri plate. *E. coli* CCCD-E003 suspension (100 µL) was added to 6 of the 12 groups, to produce a final bacterial concentration of 10^5^ CFU/mL, and allowed to dry. To the other 6 groups, one added the same volume of TSB. All groups were incubated for 1 h at 37 °C. From the 6 groups of uteri artificially contaminated with *E. coli* CCCD-E003, 3 were inoculated with the phage cocktail at a MOI of 10 (or at a MOI of 100) (bacteria + phage—BP) and the remaining 3 groups were not inoculated with phage cocktail (bacterial control—BC). Regarding the 6 groups of non-contaminated square uteri samples with *E. coli* CCCD-E003, 3 were inoculated with the phage cocktail at a MOI of 10 (or at a MOI of 100) (phage control—PC) and the remaining 3 groups were not inoculated with the phage cocktail (uterus control—UC). To maintain a moist surface on the uterus samples and prevent dehydration, the Petri plates (60 mm ϕ) containing the uterus samples were placed inside larger Petri plates (90 mm ϕ) containing 10 mL of sterile PBS. All 12 groups were incubated precisely under the same conditions. The uterus test samples and controls were sampled at times 0, 1, 2, 3, 4, 5, 6, 8, 10 and 12 h. For every sampling time, the sampled uterus squares were placed in 10 mL of sterile PBS and incubated for 30 min at 37 °C with orbital shaking (130 rpm), to allow desorption of both bacterial cells and phage particles. The phage titer of the supernatant was determined in triplicate for all assays via the double-layer agar method, after 18 h of incubation at 37 °C, whereas the bacterial concentration was determined in triplicate via the drop-plate method in solid TSA, after 24 h of incubation at 37 °C. This ex vivo assay was repeated three times in different periods in time, to ensure independent replicates. A simplified scheme of the procedure followed is depicted in Figure 1.

### 2.15. Purification of Phage DNA and Whole Genome Sequencing

Purified DNA samples of the isolated phages were sequenced at NGS SOLUÇÕES GENÔMICAS (Piracicaba/SP, Brazil) using the Illumina MiSeq platform. PEG-concentrated phage suspensions (500 µL) were treated with DNAse-I, RNAse and Proteinase K, as described elsewhere [36,46]. Phage DNA extraction was then performed via the phenol: chloroform methodology described in detail elsewhere [36,46]. The purity and concentration of the extracted phage DNA were determined in a DS-11FX spectrophotometer (DeNovix Inc., Wilmington, DE, USA) at 260 nm, 280 nm and 230 nm. Before preparation of the shotgun genomic libraries, the phage DNA was quantified in a Quantus™ Fluorometer (Promega, Madison, WI, USA).

Pure phage DNA (2–20 ng) was used to prepare the shotgun genomic library utilizing the Illumina Nextera DNA library preparation kit (Illumina, San Diego, CA, USA), and the DNA fragment library was cleaned up with Agencourt AMPure XP beads (Beckman Coulter, Indianapolis, IN, USA). The average fragment size (400–700 bp) was verified by running in a 2100 Bioanalyzer using a Agilent High Sensitivity DNA chip (Agilent, Palo Alto, CA, USA). Quantification of Illumina sequencing library via quantitative PCR (qPCR (KAPA Library Quantification Kit from Roche (Basel, Switzerland)) followed, aiming at determining the concentration (nM) so as to perform correct dilution to upload in the sequencer), clusterization, normalization, and sequencing (NextSeq 2000 (Illumina) 2 × 100 pb, average covering of 10 million clusters per genome sample) was carried out following standard sequencing protocols in Illumina MiSeq platform. The library was subjected to one run using the MiSeq Reagent kit v3 (600-cycle format, paired-end (PE) reads).

### 2.16. Phage Genome Assembly, Taxonomic Evaluation, Annotation, and Comparisons

Phage genome assembly. The sequencing reads were processed and the genome was assembled using Shovill 1.1.0 (https://github.com/tseemann/shovill (accessed on 5 September 2022)), a pipeline specialized in assembling prokaryotic genomes. In order to achieve fast and accurate assembly, Shovill performs several steps using various bioinformatics tools: (i) estimates genome size using KMC version 3.1.1 [57]; (ii) reduces the number of sequencing reads to get around 250× coverage over estimated genome size; (iii) remove Illumina adapters with Trimmomatic version 0.39 [58]; (iv) fixes bugs in sequencing reads using Lighter version 1.1.2 [59]; (v) join paired readings that overlap, with FLASH version 1.2.11 [60]; (vi) assembles the draft genome with SPAdes version 3.15.4 [61]; (vii) maps sequencing reads into draft genome assembled with BWA MEM version 0.7.17 [62]; (viii) with the mapping obtained in the previous step, correct inaccuracies in the assembly using Pilon version 1.23 [63]; and (ix) removes very short contigs (minimum size of 500 base pairs), with very low coverage, or formed exclusively by homopolymers.

Taxonomic evaluation. The taxonomic evaluation of contigs was performed with Kraken2 version 2.1.2 [64], a taxonomic classifier based on k-mers, using the PlusPF database version 20,210,517 (available at https://benlangmead.github.io/aws-indexes/k2 (accessed on 5 September 2022)). Viral contigs were separated according to the genus identified by Kraken2, Ccfind version 1.4.5 [65] was used to identify circular contigs and remove terminal repeats from circular contigs, and BLAST+ version 2.13.0 [66] was used to determine the bacteriophage most similar to the largest circular contig. To assess the synteny between the bacteriophages identified in this analysis and the closest available in the databases (identified by the BLAST+ searches described above), alignments of the entire contigs and of the predicted proteins were performed between the bacteriophages. Alignment between contigs was conducted with minimap2 version 2.24 [67], and the visualization was generated with D-GENIES version 1.3.1 [68]. Alignment between proteins was conducted with Clinker version 0.0.24 [69], and before alignment the order of proteins was reversed.

Phage genome annotation. The genome was annotated with the Bakta annotation pipeline version 1.5 [70], that combines several tools to make the prediction and obtain a functional annotation of several categories of genes: (i) transfer RNAs and transfer-messenger RNAs, with tRNAscanSE version 2.0.9 [71] and ARAGORN version 1.2.38 [72]; (ii) non-coding RNAs and ribosomal RNAs, with Infernal version 1.1.2 [73] and HMMs derived from Rfam version 14.1 [74]; (iii) CRISPR clusters, with PILER-CR version 1.0.6 [75]; (iv) protein-coding genes were predicted with Prodigal version 2.6.3 [76] and annotated with similarity searches using DIAMOND version 2.0.15 [77] against custom versions of ISfinder [78], NCBI Bacterial Antimicrobial Resistance Reference Gene Database [79] and UniProtKB (SwissProt) databases [80,81]; (v) coding genes not annotated in the previous step were annotated with HMMER version 3.2.1 [82] against the database UniprotKB/HAMAP [83]. Circular maps of the annotated phage genomes were then generated using the program CGview (version 2.0.2) [84].

### 2.17. Formulation of the Vaginal Eggs Integrating the Phage Cocktail

For the preparation of the formulation used in the manufacture of vaginal eggs, the components listed in Table 2 were used. Ten g of cocoa butter was melted at a temperature of ca. 37 °C and then 40 mg of Tween 80 was incorporated with stirring until a homogeneous mixture was obtained. One hundred mg of the preservative mixture was then added and, finally, 9 µL of each phage suspension was added, with gentle agitation. After obtaining a homogeneous mixture, pipettes for canine artificial insemination were used to aspirate the mixture, thus functioning as a template for the vaginal eggs, and subsequently stored at a temperature of 4 °C to allow the mixture to solidify. Then, the solidified mixture was removed from the mold and fractionated into 40 portions of 20 mm in length, with each solidified portion (vaginal egg) presenting a volume of 150 µL and containing 1 × 10^8^ total phage particles. The vaginal eggs thus produced were kept refrigerated at 4 °C until use was in order.

### 2.18. Evaluation of the Maintenance of Lytic Activity of the Phage Particles Integrated in the Vaginal Eggs

To check if the phage particles integrated into the vaginal egg formulation maintained their lytic viability, a sample of the formulation was cut longitudinally and placed onto the center of a bacterial lawn of *E. coli* CCCD-E003. The same procedure was independently repeated with a vaginal egg formulated without integrating the phage cocktail. Following incubation at 25 °C during 24 h, a macroscopical analysis was carried out to observe (or not) the presence of clear zones of lysis in the bacterial lawn surrounding the vaginal egg samples.

### 2.19. Physicochemical Characterization of the Vaginal Eggs Integrating the Phage Cocktail

For the physicochemical characterization of the vaginal egg formulations produced according to the formulations depicted in Table 2, a wide array of analyses was performed, encompassing XRF, DSC, mechanical (hardness, compressibility, adhesiveness and cohesiveness) and SEM analyses.

Analyses via Energy Dispersion X-ray fluorescence (EDXRF). The vaginal egg formulations were analyzed for their elemental composition in a EDXRF system (Malvern Panalytical, Santo Amaro/SP, Brazil), encompassing a Silicon Drift Diode detector (area of 25 mm^2^ and thickness of 500 μm) protected by a beryllium window of 12.5 μm. The source of X-rays utilized to excite the samples was a miniature X-ray tube with an Ag anode, programmed to work on four different excitation energies and different filters were used in front of the X-ray detector (E_1_ = 10 kV (no filter), E_2_ = 12 kV (with Al filter), E_3_ = 50 kV (with Cu filter) and E_4_ = 50 kV (with Ag filter)). The detection system possessed an energy resolution of ca. 125 eV (full width at half maximum, FWHM) for Mn Kα 5.9 keV X-rays from a ^55^Fe source. All determinations were performed using ambient air, with a total measuring time of 45 min for each sample. Signal control and data acquisition were carried out via software Epsilon version 1.7.F (8.35 B) (Malvern Panalytical B.V., Almelo, The Netherlands). While control samples were used to calibrate the EDXRF system, sample tablets (ca. 5 mm thick × 22 mm diameter) were prepared for the analyses.

Microcalorimetric analyses via DSC. Thermal characterization of the vaginal egg formulations was carried out in a differential scanning calorimeter (DSC) from Shimadzu (model DSC-60, Kyoto, Japan) together with a Thermal Analyzer (model TA 60 W) also from Shimadzu, following the procedure described elsewhere [85,86], using sample amounts in the range 1.380–1.700 mg in high-pressure aluminum pans duly sealed by pressure and a reference aluminum pan with plain air sealed inside.

Mechanical analyses. The mechanical features (compressibility, hardness, cohesiveness and adhesiveness) of the vaginal egg formulation integrating the lytic phage cocktail were determined from the relationship between force (N) versus time (s) in a texturometer from Stable Micro Systems (model TA.XT Plus Texture Analyser, Godalming, United Kingdom), produced by a probe (P/10). Beforehand, the equipment was calibrated with a load cell (mass of 5 kg). At a room temperature of 19 °C, the analytical probe (10 mm diameter) was compressed on the surface of the vaginal egg sample (rate of 0.5 mm s^−1^, with a 2.5% strain mode), with a force of 0.005 N, and with the penetration depth set to 1 mm. This procedure was performed in triplicate.

Scanning Electron Microscopy (SEM) analyses. Following lyophilization (ThermoFisher, model ModulyOD R23T-659559-RT, Pittsburgh, PA, USA), the surface and internal morphology of the vaginal egg formulation’s cocoa butter matrix integrating the lytic phage cocktail was inspected in a scanning electron microscope (JEOL, model JSM-IT200, Tokyo, Japan) working at ultra-high vacuum. Samples of lyophilized vaginal egg were cryo-fractured following deep freezing at −86 °C, fixed on a carbon layer and sputter-coated with an Au film (92 Å thickness) via cathodic pulverization produced by evaporation in a metalizing device also from JEOL (Sputter Coater model DII-29010SCTR Smart Coater, Tokyo, Japan). Photomicrographs were obtained using electron beams with acceleration speeds of 3.0 keV.

### 2.20. Evaluation of the Cytotoxicity Potential of the Vaginal Eggs Integrating Both Phage Particles, via the Agar Disk-Diffusion Assay

The cytotoxicity potential of samples of the vaginal egg formulation integrating the cocktail of lytic phage particles was evaluated via the agar disk-diffusion methodology using two cell lineages, viz. HaCaT (human keratinocytes) and V79 (mouse lung), following the procedure described elsewhere [85,86]. Inspection of the inoculated plaques was carried out macroscopically, with formation of a clear halo around the tested sample, due to cell lysis, indicating cytotoxicity [87], and microscopically, for the morphological changes of the cells surrounding the sample [47,86,88,89].

### 2.21. Statistical Analyses

**Figure 2:**
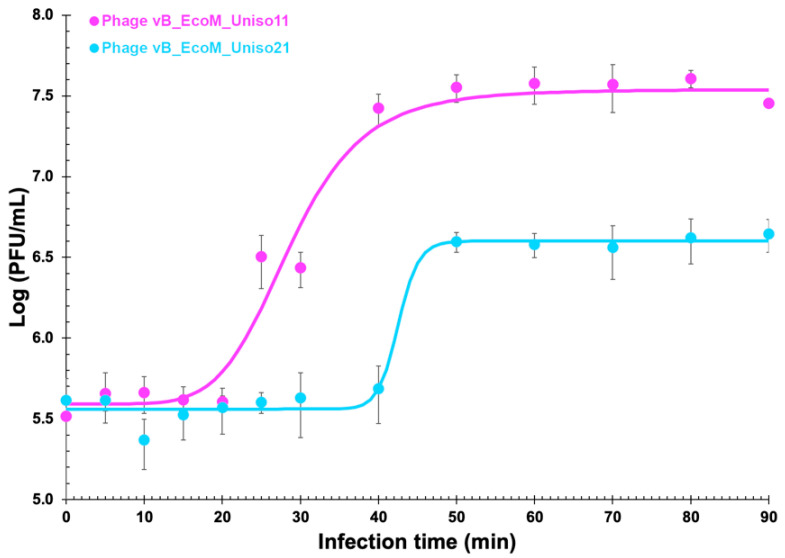
One-step growth curve analysis of phages vB_EcoM_Uniso11 and vB_EcoM_Uniso21 on an exponential culture of *E. coli*. Solid lines represent the non-linear fittings of the 4-PL model to the experimental data. Values are the means of three independent assays; Error bars represent the standard deviations.

**Figure 3:**
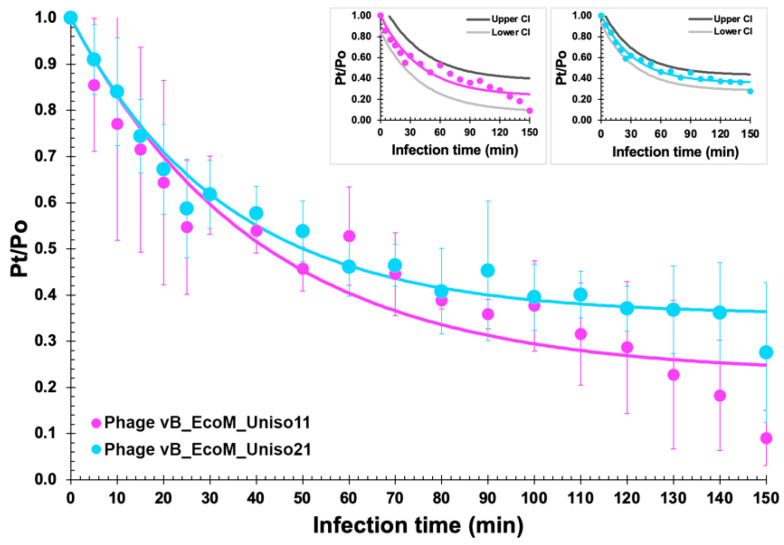
Adsorption curves of phages vB_EcoM_Uniso11 and vB_EcoM_Uniso21 particles onto *E. coli* CCCD-E003 host cells, allowing to calculate the phage particles adsorption rate following non-linear fitting of the logarithmic function to the experimental data. Values are the means of three independent assays; error bars represent the standard deviations.

For the statistical analyses of the goodness of the non-linear fittings of the 4-PL and adsorption mathematical models performed to the experimental data, tests of lack of fit of the expectation functions were carried out with respect to the phage growth (Figure 2) and adsorption (Figure 3) data, respectively. Since the subspace containing the replications of the data is orthogonal to the subspace containing not only the averages but also the expectation function [51,90,91,92], the lack of fit analyses involve comparing the ratio of the lack of fit mean square (SS_lack of fit_/NDF_lack of fit_) over the replications mean square (SS_replications_/NDF_replications_) (i.e., the *F*-ratio) with the appropriate value in the *F*-table (F(ν_NDF_ lack of fit; ν_NDF_ replications; α = 5%)).

**Figure 4:**
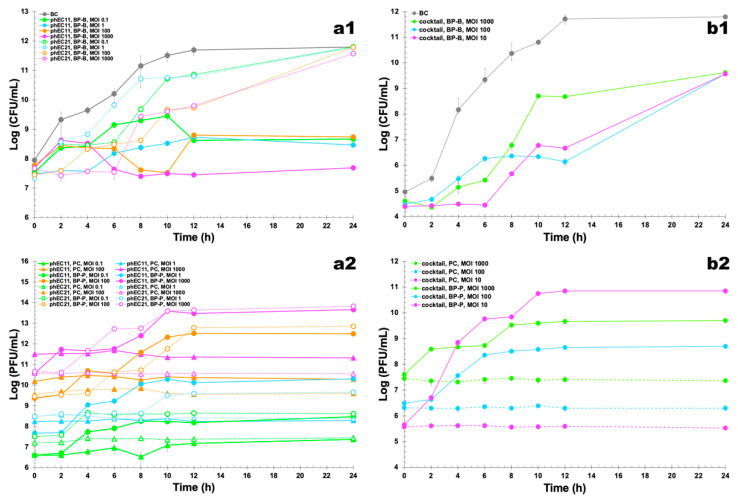
In vitro inactivation of *E. coli* by (**a1**) independent phages vB_EcoM_Uniso11 and vB_EcoM_Uniso21 at a multiplicity of infection (MOI) of 0.1, 1, 100 and 1000, and by (**b1**) their cocktail at MOI values of 10, 100 and 1000 during 24 h. (**a1**,**b1**) Bacterial concentration: BC, bacterial control; BP-B, bacteria with phage; (**a2**,**b2**) phage concentration: PC, phage control; BP-P, bacteria with phage. Values represent the mean of three independent assays whereas error bars represent the standard deviation.

**Figure 5:**
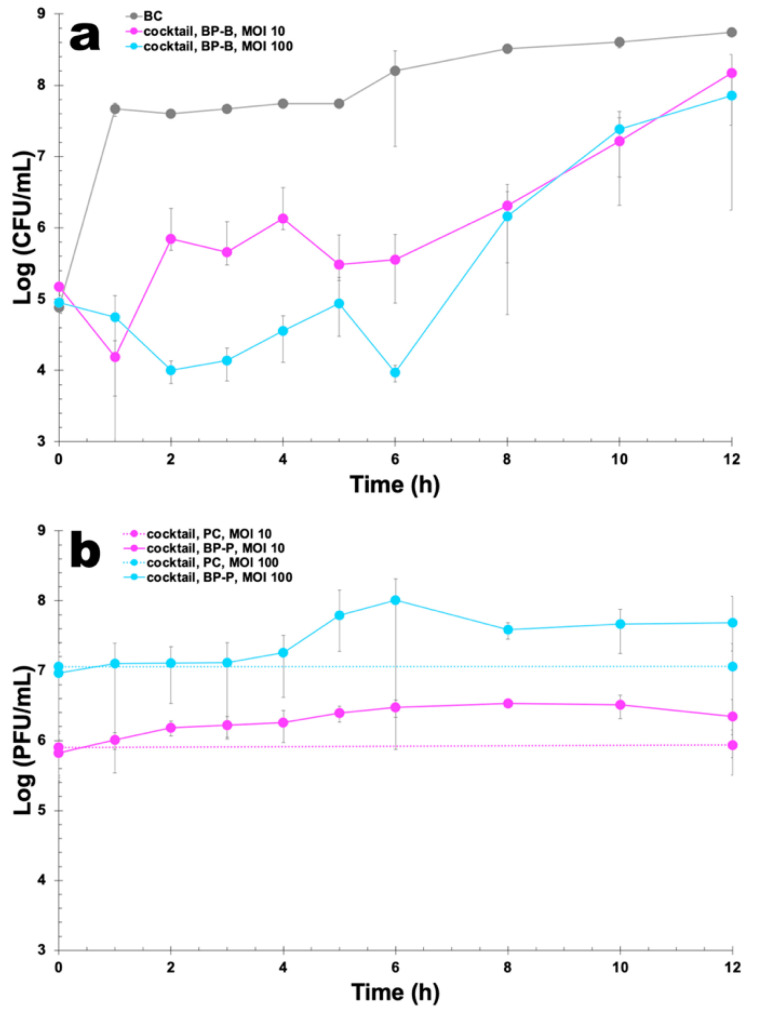
Ex vivo inactivation of *E. coli* in artificially contaminated canine uterus by phage cocktail at MOI values of 10 and 100, during 12 h. (**a**) Bacterial concentration: BC, bacterial control; BP-B, bacteria with phage; (**b**) phage concentration: PC, phage control; BP-P, bacteria with phage. Values represent the mean of three independent assays whereas error bars represent the standard deviation.

The experimental data produced in in vitro and ex vivo phage-bacteria inactivation assays was statistically analyzed with GraphPad Prism 7.04 (GraphPad Software, San Diego, CA, USA). While normal distribution of the data was checked by Kolmogorov-Smirnov test, homoscedasticity was checked by Levene’s test. Significance of bacterial and phage concentrations in in vitro and ex vivo phage-bacteria inactivation assays, throughout the sampling times, was tested using two-way ANOVA and Bonferroni *post hoc* tests. The significance of the differences registered for bacterial concentrations (Figure 4a1,b1 and Figure 5a) was assessed via comparison of the results of the test samples for each experimental treatment (*E. coli*, BP) with the corresponding bacterial control (*E. coli*, BC), for the different incubation times, whereas the significance of the differences registered for viral concentrations (Figure 4a2,b2 and Figure 5b) in the bacterial inactivation assays was assessed via comparison of the results obtained in the test (*E. coli*, BP) with the corresponding phage control (PC), for the different sampling times. The significance of the differences between the different MOI tested was checked by comparing the results obtained in the test samples (*E. coli*, BP) with the results obtained for the control samples (*E. coli*, BC), for the different inactivation times. For testing the significance of the interactions for the bacterial concentrations (Figure 4a1,b1 and Figure 5a), the *F*-test ((MS_bacterial density_)/(MS_interaction density_ × treatment; (MS_sampling time_)/(MS_interaction time_ × treatment)) was also applied. A value of *p* < 0.05 was considered to be statistically significant.

## 3. Results

In the research work described herein, structural and functional stabilization of two newly isolated virulent phage particles for *E. coli* within a vaginal egg-type formulation has been proposed, aiming at intrauterine bacterial control in veterinary settings. The two isolated phages were amplified in a *E. coli* CCCD-E003 bacterial strain and, to assess their potential for veterinary phage therapy, extensive physicochemical, biological and molecular characterization was entailed.

### 3.1. One-Step Growth Curve Analysis

Non-linear fitting of the one-step growth data to a 4-PL model showed that the eclipse period, latent period and intracellular accumulation period lasts 20, 40 and 20 min for phage vB_EcoM_Uniso11, respectively (Figure 2). Phage vB_EcoM_Uniso21 growth produced eclipse, latent and intracellular accumulation periods of 36, 48 and 12 min, respectively. While the burst size of phage vB_EcoM_Uniso11 was 106 PFU/host cell, the burst size of phage vB_EcoM_Uniso21 was 10 PFU/host cell.

### 3.2. Adsorption Curve

Approximately 30% and 25% of the phage particles adsorb to *E. coli* CCCD-E003 cells after 30 min, 50% and 45% adsorbed after 60 min, and 90% and 75% adsorbed after 150 min, respectively (Figure 3), for phages vB_EcoM_Uniso11 and vB_EcoM_Uniso21.

Nonlinear fitting of the experimental data to the logarithmic model, allowed the determination of the adsorption rates of the phage particles: δ_vB_EcoM_Uniso11_ = 1.904 × 10^−10^ PFU^−1^ CFU^−1^ mL^−1^ h^−1^ (ϕ_vB_EcoM_Uniso11_ = 5.664 × 10^−3^ mL min^−1^; X_0_ = 1.0 × 10^8^ CFU mL^−1^; r^2^ (coefficient of determination) = 0.9125) and δ_vB_EcoM_Uniso21_ = 1.926 × 10^−10^ PFU^−1^ CFU^−1^ mL^−1^ h^−1^ (ϕ_vB_EcoM_Uniso21_ = 1.070 × 10^−2^ mL min^−1^; X_0_ = 1.0 × 10^8^ CFU mL^−1^; r^2^ (coefficient of determination) = 0.9713).

Since the standard deviations produced for the experimental adsorption data were relatively high, a statistical analysis of lack of fit of the exponential adsorption decay model [55,56] was performed. At 0.05 (95% confidence), no lack of fit was noticed for the adsorption data of the two phages (phage vB_EcoM_Uniso11: calculated *F*_ratio_ = 0.1921, standard *F*_ratio_ = 2.0147, *p*-value = 0.9990; phage vB_EcoM_Uniso21: calculated *F*_ratio_ = 0.0517, standard *F*_ratio_ = 2.0147, *p*-value = 0.9999). Because “lack of fit” is the fluctuation of the experimental data around the fitted (mathematical) model, a *p*-value > 0.10 (a statistically insignificant lack of fit), as was in fact determined, means that the model fits/predicts the experimental response data. To further make this conclusion more noticeable, small plots were inserted in Figure 3, containing upper and lower 95% confidence intervals of the non-linear fittings performed to the phage adsorption data.

### 3.3. In Vitro Phage Assays

Bacterial concentration in the control (BC) increased by 3.9 log CFU/mL (ANOVA, *p* < 0.05) during the 24 h of incubation (Figure 4a1). When using the two phages in an independent fashion, increasing the MOI from 0.1 to 1000 did significantly increase (ANOVA, *p* < 0.05) bacterial inactivation after 6 h (from 1.05 log CFU/mL to 2.57 log CFU/mL (phage vB_EcoM_Uniso11) and from 1.64 log CFU/mL to 2.66 log CFU/mL (phage vB_EcoM_Uniso21)), 10 h (from 2.06 log CFU/mL to 4.01 log CFU/mL (phage vB_EcoM_Uniso11) and from 0.79 log CFU/mL to 1.90 log CFU/mL (phage vB_EcoM_Uniso21)) and by 12 h (from 3.07 log CFU/mL to 4.24 log CFU/mL (phage vB_EcoM_Uniso11) and from 0.83 log CFU/mL to 1.90 log CFU/mL (phage vB_EcoM_Uniso21)) (Figure 4a1). At a MOI of 0.1, 1, 100 and 1000, the maximum *E. coli* CCCD-E003 inactivation with phage vB_EcoM_Uniso11 was 3.07, 2.96, 2.89 and 4.24 log CFU/mL, respectively (Figure 4a1, ANOVA, *p* < 0.05), obtained after an incubation timeframe of 12 h, when comparing with those concentrations for the BC. Changing the MOI from 0.1 to 1 did not promote a significant increase in bacterial inactivation after 12 h of incubation (ANOVA, *p* > 0.05), considering both phages separately (Figure 4a1). When using phage vB_EcoM_Uniso11, inactivation was identical for MOI 100 (3.99 log CFU/mL) and 1000 (4.01 log CFU/mL) (ANOVA, *p* > 0.05), along the first 10 h of phage-bacteria incubation. However, after 12 h of phage-bacteria incubation, inactivation was higher for MOI 1000 (by a factor of 1.47×, viz. 4.24 log CFU/mL, ANOVA, *p* < 0.05). When using phage vB_EcoM_Uniso21 at MOI 1000, the maximum of bacteria inactivation was 2.66 log CFU/mL (Figure 4a1, ANOVA, *p* < 0.05), attained after 6 h of phage-bacteria incubation (ANOVA, *p* < 0.05). However, increasing MOI from 1 to 1000 did significantly increase bacterial inactivation (1.90 log CFU/mL) after 12 h (Figure 4a1, ANOVA, *p* < 0.05). Despite this, for phage vB_EcoM_Uniso21, after the first 10 h of incubation, bacterial inactivation was identical for MOI 100 (1.85 log CFU/mL) and 1000 (1.90 log CFU/mL) (ANOVA, *p* > 0.05). When using phage vB_EcoM_Uniso11, bacterial inactivation at MOI 1000 (4.24 log CFU/mL) was significantly higher (ANOVA, *p* < 0.05) than that produced with MOI 100 (2.89 log CFU/mL) after 12 h of incubation. Bacterial inactivation by phages vB_EcoM_Uniso11 and vB_EcoM_Uniso21 at MOI 1000 was statistically different during the treatment (ANOVA, *p* < 0.05). With phage vB_EcoM_Uniso11 at MOI 1, bacterial inactivation (2.96 log CFU/mL) was, in general, significantly higher (ANOVA, *p* < 0.05) than that produced by phage vB_EcoM_Uniso21 (0.89 log CFU/mL). When using phage vB_EcoM_Uniso11 at MOI 0.1, 1 and 1000, no regrowth of bacteria whatsoever was observed after 10 h of phage-bacteria incubation until the end of the treatment timeframe, but this trend was reversed when using phage vB_EcoM_Uniso21, with a significant bacterial regrowth being observed at all MOI after 8 h of incubation (ANOVA, *p* < 0.05) (Figure 4a1). Despite this, by the end of the phage-bacteria incubation timeframe the bacterial densities in the different treatments with phage vB_EcoM_Uniso11 were significantly lower than those observed for the bacterial control (BC, Figure 4a1).

Regarding phage virion concentrations, while the controls (PC) remained fairly constant throughout the phage-bacteria incubation timeframe (ANOVA, *p* > 0.05), incubation of phage vB_EcoM_Uniso11 with its bacterial host promoted a statistically significant increase in the virion concentration of this phage (ANOVA, *p* < 0.05), viz. 2.63 log PFU/mL (MOI 1), 3.13 log PFU/mL (MOI 100) and 3.08 log PFU/mL (MOI 1000) (Figure 4a2). The same trend was observed for phage vB_EcoM_Uniso21, which showed a statistically significant increase in phage virion concentration (ANOVA, *p* < 0.05) upon incubation with its bacterial host, viz. 3.40 log PFU/mL (MOI 100) and 3.16 log PFU/mL (MOI 1000) (Figure 4a2).

Throughout the 24 h timeframe of the experiments, the concentration of phage vB_EcoM_Uniso11 in the controls (PC) showed a slight increase at MOI 0.1 (viz. 0.84 log PFU/mL, ANOVA, *p* > 0.05) (Figure 4a2).

When used as a cocktail, incubated in the presence of its bacterial host, a statistically significant decrease in bacterial concentration was observed for MOI 10 (Figure 4b1) after 6 h (4.90 log PFU/mL, ANOVA, *p* < 0.05), 8 h (4.69 log PFU/mL, ANOVA, *p* < 0.05) and 12 h (5.05 log PFU/mL, ANOVA, *p* < 0.05) of treatment. Increasing the MOI from 100 to 1000 did not promote significant changes in bacterial reduction (3.92 log PFU/mL, ANOVA, *p* > 0.05). During the first 12 h of treatment, the concentration of bacteria was significantly reduced at MOI 10 (5.05 log CFU/mL, ANOVA, *p* < 0.05) and 100 (5.57 log CFU/mL, ANOVA, *p* < 0.05) (Figure 4b1). After 12 h of treatment, increasing the cocktail MOI from 10 to 100 did not promote significant changes in bacterial reduction (5.05 to 5.57 log PFU/mL, ANOVA, *p* > 0.05) and, in addition, after the first 12 h of treatment, a significant bacterial regrowth was observed until the end of the incubation timeframe (ANOVA, *p* < 0.05) (Figure 4b1) for all MOI values studied.

Throughout the entire phage-bacteria incubation timeframe, whilst the phage controls (PC) persisted fairly constantly (ANOVA, *p* > 0.05), when the two-phage cocktail was incubated with its bacterial host, a statistically significant increase in the virion concentration was noticed (ANOVA, *p* < 0.05), viz. 5.19 log PFU/mL (MOI 10), 2.21 log PFU/mL (MOI 100) and 2.09 log PFU/mL (MOI 1000) (Figure 4b2).

### 3.4. Ex Vivo Phage Treatment Experiments in Artificially Contaminated Canine Uterus

*E. coli* density in the BC increased by 3.86 log CFU/mL (Figure 5) during the 12 h timeframe of incubation. The statistical ANOVA performed revealed that the differences between sampling time among treatment of *E. coli* with the phage cocktail (*p* < 0.05) and the differences between density among treatment of the *E. coli* (*p* < 0.05) were significant when comparing the two MOI values. The phages in the phage cocktail infected and killed *E. coli* cells in the artificially contaminated canine uterus. The maximum of *E. coli* inactivation was 1.75 and 3.60 log CFU/mL, for MOI 10 and 100, respectively (Figure 5a, Bonferroni, *p* < 0.05), after 2 h of incubation, whereas the maximum of *E. coli* inactivation was 2.65 and 4.23 log CFU/mL, for MOI 10 and 100, respectively (Figure 5a, Bonferroni, *p* < 0.05), after 6 h of incubation. After 8 h of incubation, the *E. coli* counts (reduction of 2.20 and 2.35 log CFU/mL for MOI 10 and 100, respectively) were statistically similar (Bonferroni, *p* > 0.05) when compared to the values obtained in the bacterial control. After 12 h of incubation, the *E. coli* counts (reduction of 0.57 and 0.89 log CFU/mL for MOI 10 and 100, respectively) were also statistically similar (Bonferroni, *p* > 0.05) when compared to the values obtained in the bacterial control (Figure 5a). After the first 8 h of treatment, a significant bacterial regrowth was observed until the end of the incubation timeframe (ANOVA, *p* < 0.05) (Figure 5a) for both MOI values studied. While phage controls (PC) remained fairly constant throughout the experiments (ANOVA, *p* > 0.05), when the phage cocktail was incubated in the presence of planktonic bacterial host cells a statistically significant increase (0.53 and 0.72 log PFU/mL, Bonferroni, *p* < 0.05) was observed for MOI 10 and 100, respectively (Figure 5b).

**Figure 6:**
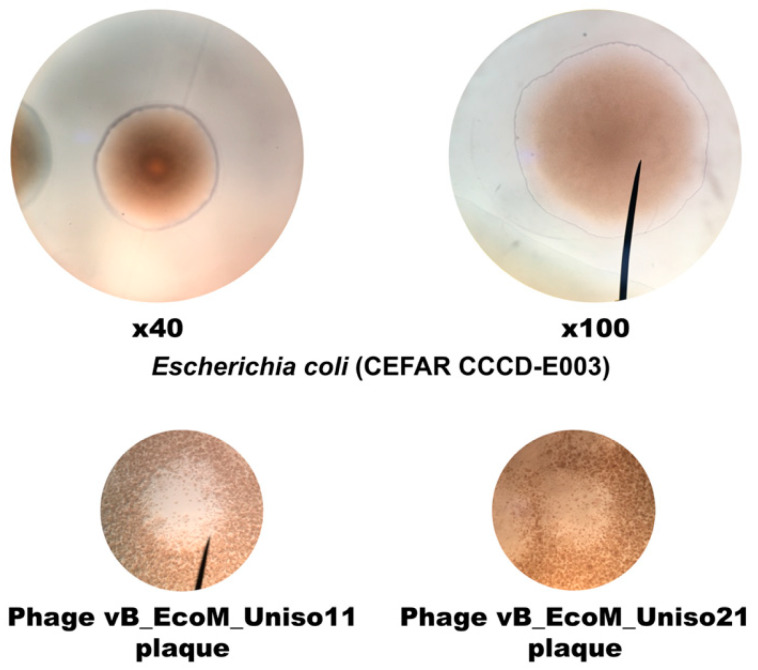
Morphological characteristics of *E. coli* CCCD-E003 host colonies on solid TSA, allowing for the observation of cell swimming motility characteristics, and of phages vB_EcoM_Uniso11 and vB_EcoM_Uniso21 plaques of lysis on a bacterial host lawn.

Figure 6 displays the morphology of *E. coli* CCCD-E003 host cell colonies on solid TSB and of phages vB_EcoM_Uniso11 and vB_EcoM_Uniso21 plaques of lysis on a bacterial host lawn, under optical microscopy.

**Figure 7:**
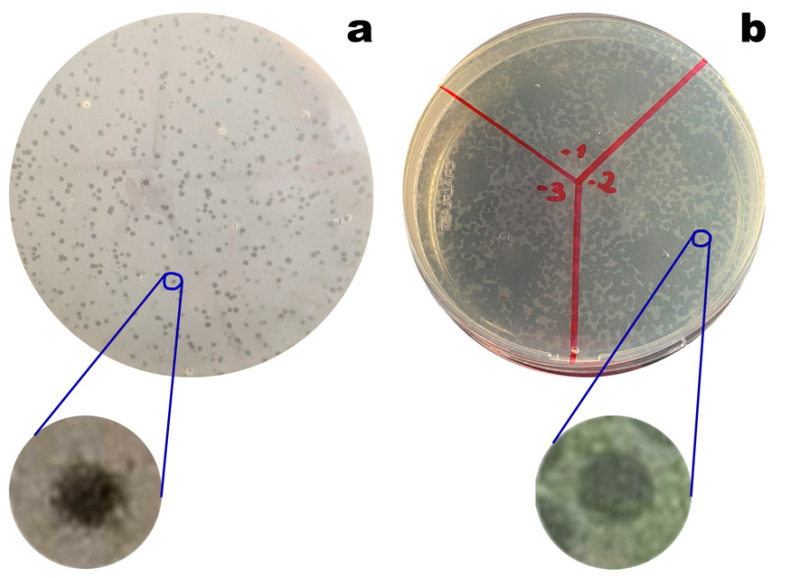
Morphology of phages vB_EcoM_Uniso11 (**a**) and vB_EcoM_Uniso21 (**b**) plaques on their bacterial host (*E. coli* CCCD-E003). Lysis plaques are zoomed out, to observe the absence of secondary halos.

*E. coli* cells display two flagella-driven motility types, swimming (movement of individual cells in either liquid medium or semisolid soft agar) and swarming (collective cellular movement on semisolid agar) [93]. As can be observed in Figure 6, the *E. coli* cells exhibited swimming motility. The phage plaques on the bacterial host lawn were tiny and displayed diameters ranging from ≈0.5 to ≈1 mm (Figure 7).

### 3.5. Phage Plaque Isolation

Phages vB_EcoM_Uniso11 and vB_EcoM_Uniso21, isolated from sewage water of the Veterinary Hospital at UNISO (Sorocaba/SP, Brazil), formed dimensionally different and clear plaques on the *E. coli* CCCD-E003 lawns, with diameters ranging from ≈0.5 to ≈1 mm (Figure 7). No secondary halo could be observed in the frontier of the lysis plaques of phages vB_EcoM_Uniso11 (Figure 7a) and vB_EcoM_Uniso21 (Figure 7b), indicative of the absence of production of phage depolymerases [94,95] by these phages (zoomed out plaques). High titer suspensions (10^11^ PFU/mL) were obtained for the two phages.

### 3.6. Isolation and Characterization of Bacterial Strains Responsible for Animal Pyometra Infections

The results obtained in the biochemical tests performed to the bacterial isolates originating from veterinary clinical samples are displayed in Table 3. Biological samples were collected from uteri of canine and cat females previously diagnosed with (or suspected of) uterine infection, and from uteri of mares.

From the inoculum of clinical isolates, plating was performed in solid TSA followed by overnight incubation at 37 °C. Sample A2, being Gram-positive (Table 3), was not tested via Enterokit B, since it is not an Enterobacterium. The catalase test was performed, which was negative, and its morphology was microscopically observed as cocci in a chain arrangement and then identified as *Streptococcus* sp. Regarding sample A12, it did not show bacterial growth. The analyzes performed on the samples obtained in this study are in accordance with the literature, indicating the prevalence of *E. coli* bacteria in clinical isolates from uterine infections known as pyometra, very common in female dogs and non-castrated cats [24,25,26,27,96], and also in mares.

### 3.7. UV-Vis Spectral Scans for Determination of the Phage Particles Molar Extinction Coefficients

**Figure 8:**
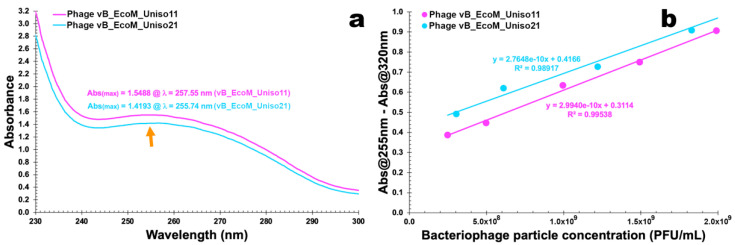
UV-Vis spectral scans of dilutions of the concentrated phage suspensions (**a**) and linear relationships between the concentration of (whole) phage particles and absorption at 255 nm corrected for cell debris and other cytoplasmatic proteins at a wavelength of 320 nm (**b**).

The results gathered from UV-Vis spectral scans performed to the PEG-concentrated phages vB_EcoM_Uniso11 and vB_EcoM_Uniso21 can be found in Figure 8a, whereas the data utilized to prepare the linear relationships between phage particle concentration and phage corrected absorbance (Figure 8b) are exhibited in Supplementary Table S1.

For both phages, a maximum absorption was noticed around 255 nm and a minimum absorption was noticed around 245 nm (indicative of the absence of bacterial cell debris and presence of a high number of phage virions). The (linear) Beer-Lambert equation was then fitted to the experimental data displayed in Supplementary Table S1, allowing to determine the molar extinction coefficients of the isolated phages as ε_vB_EcoM_Uniso11_ = 2.9940 × 10^−10^ (PFU/mL)^−1^.cm^−1^ and ε_vB_EcoM_Uniso21_ = 2.7648 × 10^−10^ (PFU/mL)^−1^.cm^−1^ (Figure 8). Subtracting Abs_320nm_, a wavelength where there is negligible light absorption from phage chromophores, aims at crudely correcting for light scattering from phage particles and non-phage particulate contaminants [36,46,47,49,50].

Phages are protein-based entities, with their structure encompassing major capsid, tail, baseplate, and spike proteins. In the UV wavelength range, proteins absorb radiation with a maximum absorption at 278–280 nm due to the contributions from four chromophores, viz. side chains of the amino acids tryptophan, tyrosine and phenylalanine and disulfide bonds of cysteines. The Beer-Lambert law assumes that a fraction of the light passing through a sample is absorbed and the remaining light is transmitted. For large molecules such as proteins and whole virus particles, extinction of light due to both absorption and scattering results in a deviation from the Beer-Lambert law. Hence, attenuation of the amount of light reaching the detector due to absorption by protein chromophores and scattering subtracted from light attenuation due to the presence of chromophores only, produces the absorbance characteristic for a given phage particle at a given concentration [97]. Phage structural proteins are, therefore, the major contributors for the absorption spectrum in the UV range. At a wavelength of 320 nm phage protein chromophores have virtually null absorption (although different from zero), and this absorption is used to correct for light scattering from phage particles and non-phage particulate contaminants. The optical density at 320 nm is therefore totally due to scattering [98]. The two phages isolated are approximately spherical particles, having roughly equal weight ratios of protein to DNA; their structural proteins therefore contribute substantially to the absorption spectrum, accounting for the broad plateau between 250 and 280 nm, with a shallow maximum at 258 nm (phage ph0011) or 256 nm (phage ph0021).

### 3.8. SDS-PAGE Analysis of Phage Structural Proteins

**Figure 9:**
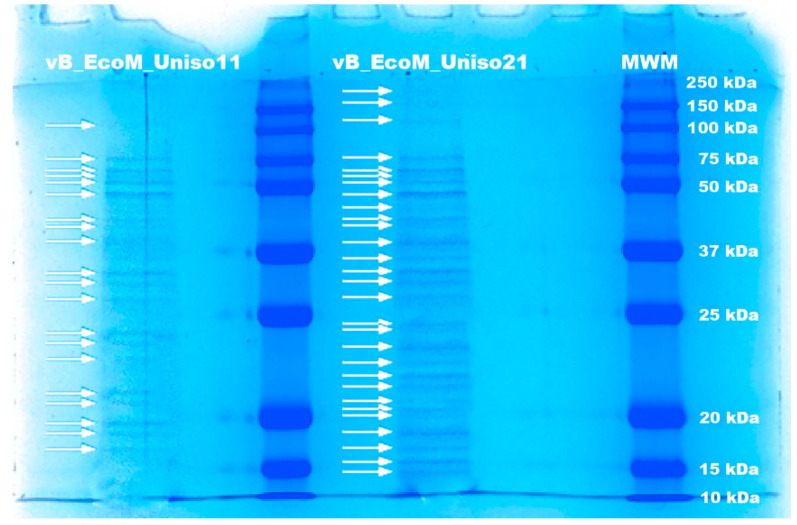
Coomassie-stained electrophoretogram of structural proteins of phages vB_EcoM_Uniso11 and vB_EcoM_Uniso21, and wide-range molecular weight markers (lane MWM).

The structural protein profile of the two phages was obtained via SDS-PAGE analysis (Figure 9), and revealed distinct protein profiles, providing a first indication that the two phages are different.

For phage vB_EcoM_Uniso11, twenty different protein bands were detected, ranging from approximately 16.57 to 127.39 kDa (Figure 9). Twenty-nine different protein bands ranging from approximately 13.49 to 235.09 kDa were detected for phage vB_EcoM_Uniso21.

### 3.9. Transmission Electron Microscopy (TEM) Analyses

**Figure 10:**
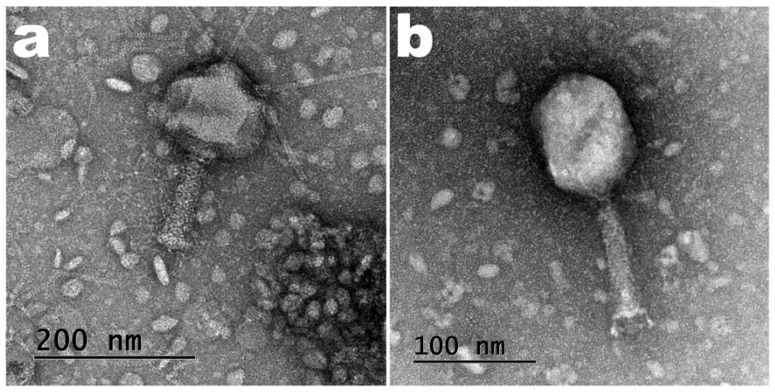
Negative-staining TEM photomicrographs of phages vB_EcoM_Uniso11 (**a**) and vB_EcoM_Uniso21 (**b**).

Undiluted samples of PEG-concentrated phage suspensions were analyzed by TEM, aiming at observing their structures. Despite the fact that the negative staining procedure of the phage particles involved quite gentle air-drying, nevertheless a slight deformation of the virion capsids was produced, as can be observed in the photomicrographs of Figure 10.

Based on the morphological analysis entailed by TEM (Figure 10), the two phages were identified as belonging to the order Caudovirales. Phages vB_EcoM_Uniso11 (ph0011) and vB_EcoM_Uniso21 (ph0021) were putatively identified as members of family *Myoviridae*. Phage ph0011 has an elongated icosahedral head with approximately 129.00 ± 3.96 nm and a long contractile tail with a length × thickness of approximately (118.49 ± 1.44) × (29.83 ± 2.65) nm. Regarding phage ph0021, it has also an elongated icosahedral head with approximately 106.72 ± 3.55 nm, and a long contractile tail with a length × thickness of approximately (101.12 ± 1.05) × (19.61 ± 2.53) nm. All phage particle measurements were performed using the freely available, open source, image processing software ImageJ (Fiji, version 2.0.0-rc-68/1.52e) from the National Institute of Health (USA). Seven particles were measured for each phage and the averages and standard deviations were calculated as displayed above.

### 3.10. Determination of Phage Particles Physical Parameters

Phage ph0011 and ph0021 suspensions possessed homogeneous particles (low polydispersity indexes) with negative Zeta potentials, indicative of the phage particle stability. Phages ph0011 and ph0021 exhibited average particle hydrodynamic sizes of ca. 311 nm and 109 nm, respectively (Table 4).

The diffusion coefficients of the phage particles were one order of magnitude above that of *E. coli* CCCD-E003 (Table 4). The phage particle measurements obtained by TEM produced average particle sizes of 247.5 nm for phage ph0011 and 207.84 nm for phage ph0021, considering the total length of the phages (head plus tail). These values, however, compare grossly with the values obtained via DLS, since DLS considers the particle in suspension to be, in average, a sphere. Although the order of magnitude of the measurements was the same for both techniques, the actual values are in fact different. Hence, we are more confident on the average particle measurements obtained by TEM analysis.

### 3.11. Host Range of the Two Newly Isolated Phages: Spot Test and Efficiency of Plating (EOP)

Spot testing indicated that phages vB_EcoM_Uniso11 and vB_EcoM_Uniso21 had the capacity to form completely cleared zones on 12 of the 19 strains from clinical isolates (Table 1). Phage vB_EcoM_Uniso11 infected *E. coli* strains from clinical isolates A1, A4, A5, A7, A8, A9, A11, A14, A17, A19, A21 and A26, whereas phage vB_EcoM_Uniso21 infected the very same strains, with variable efficiencies of plating (Table 1). Both phages also infected collection *E. coli* strains *3.2, *3.4 and ATCC-25922, The efficiency of plating of both phages was quite low for two of the clinical isolates from mares and, although producing clear halos of lysis on bacterial lawns of those strains, were probably not capable of producing a large number of virions (Table 1).

None of the two phages was effective against *S. enterica* CCCD-S004, *P. aeruginosa* CCCD-P004, *P. mirabilis* CCCD-P001, *E. faecalis* CCCD-E002, *B. subtilis* CCCD-B010, *S. epidermidis* CCCD-S010, *S. aureus* CCCD-S009, *K. pneumoniae* CCCD-K001, *Enterobacter sp.* (*2.2), *E. aerogenes* (*2.13), *K. pneumoniae* (*4.15), S. intermedius*, *Pseudomonas syringae* pv. syringae*, *Pseudomonas syringae* pv. garcae*, *Xanthomonas axonopodis* pv. *citri* 306*, *P. penneri* (*5.5) and *P. vulgaris* (*5.4) (Table 1).

### 3.12. Genomic Characterization of Phages vB_EcoM_Uniso11 and vB_EcoM_Uniso21

The genomes of phages ph0011 and ph0021 were sequenced and assembled, resulting in a contig of 348,288 bp and 165,222 bp, respectively. The two contigs were circular with overlapping of the sequences from both ends of the contig, indicating that the assembled phage genomes are complete. The GC content of the ph0011 phage genome is 45%, whereas that of the ph0021 phage genome is 43%. Overall characteristics of the assembly and of the genome annotations are summarized in Table 5.

**Figure 11:**
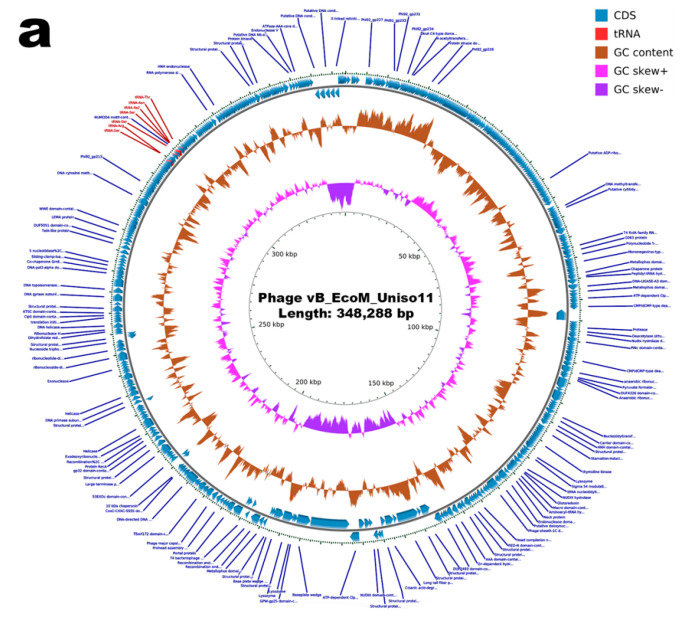

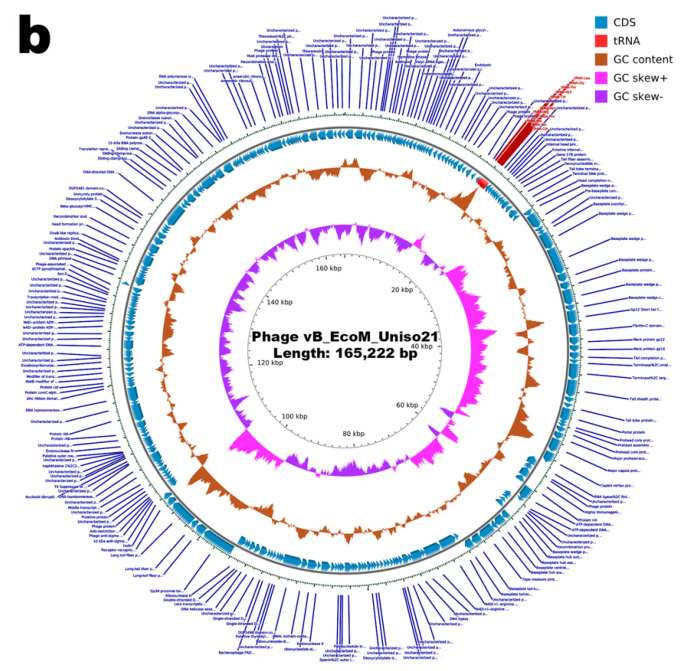
Annotated genome maps of phages ph0011 (**a**) and ph0021 (**b**). In these circular maps, predicted CDS, GC skew (+/−) and GC content are displayed. The innermost ring indicates the GC skew of phage genome (+, magenta; −, purple), the middle ring indicates GC content (brown) and, in the outer ring, the blue arrows represent the annotated coding sequences (CDSs) according to the annotation in Supplemental Table S1 and the red arrows represent tRNAs. The direction of the blue arrows represent the direction of transcription (strand + or −).

While phage ph0011 genome was found to encode 7 tRNAs and 575 protein coding sequences (CDS, or genes) (see Supplementary Table S2), phage ph0021 genome was found to encode 11 tRNAs and 264 protein coding sequences. Comparing the annotated protein coding sequences (CDS) with different databases revealed that, in the case of phage ph0011 genome, 447 CDS are predicted as hypothetical proteins (proteins of unknown function), whereas in the case of phage ph0021 genome 19 CDS are predicted as hypothetical proteins. Archetypal phage structural proteins were annotated in the two phage genomes, including capsid, tail, baseplate and spike proteins, along with DNA metabolism-related and host lysis proteins (viz. holin, endolysin and spanin). No genes related to toxins, antibiotic resistance, depolymerases, virulence factors, or integrases, were detected among the CDS with predicted functions in the genomes of phages ph0011 and ph0021 (see Supplementary Table S2). The most part of the protein coding genes identified were annotated as hypothetical proteins, i.e., with unknown function (see Supplementary Table S2). Circular, annotated maps, of the two phage genomes are displayed in Figure 11.

### 3.13. Evaluation of the Maintenance of Lytic Activity of the Phage Particles Integrated in the Vaginal Eggs

**Figure 12:**
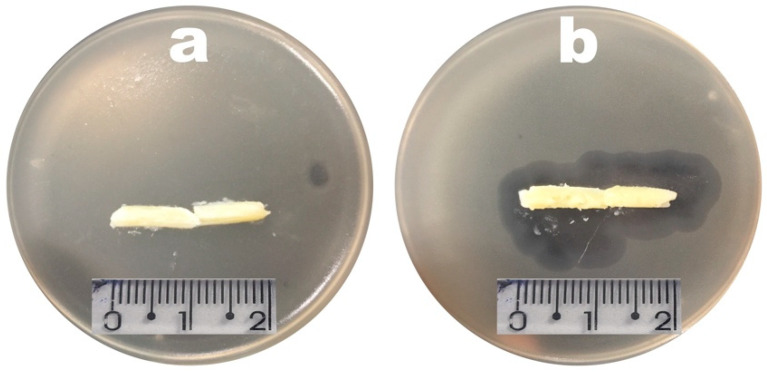
Results from evaluation of the maintenance of lytic activity of the phage particles upon integration within the bioactive cocoa butter matrix and concomitant stabilization of both their structure and function. (**a**) Cocoa butter matrix devoid of phage particles; (**b**) bioactive cocoa butter matrix integrating the cocktail of phage particles. Full scale bar represents 2 cm.

Structural and functional stabilization of the phage particles within the cocoa butter matrices was attained via entrapment of the phage particles integrating the lytic cocktail in said matrices prior to solidification of the formulation, with a uniform spatial distribution within the matrix, and the influence of the several components of the lipid matrix formulation upon lytic viability of the phage particles was assessed. Regardless of the conditions under scrutiny, all phage viability assays consisted in cutting a vaginal egg (a plain one and one with the phage cocktail) longitudinally and placing the lipid matrix (with the cut surface facing downwards) on a *E. coli* CCCD-E003 bacterial lawn, and incubation at 25 °C for 24–48 h. Following incubation, the absence or presence of lysis zones in the lawn due to the immobilized phage particles was observed. The macroscopic appearance of the bacterial lawn was examined having as control the lipid matrix devoid of phage particles. The results gathered are displayed in Figure 12.

When analyzing the two cocoa butter matrices displayed in Figure 12, no lysis zone whatsoever could be observed in the bacterial lawn where the control cocoa butter matrix was placed (Figure 12a). On the contrary, on the bacterial lawn where the cocoa butter matrix integrating the phage cocktail was placed, lysis (presence of clear zones) below and surrounding the sample was most evident (Figure 12b), allowing for the conclusion that entrapment of the phage virions within the cocoa butter matrix formulation did not interfere with their lytic activity.

### 3.14. Determination of the Elemental Profile of the Vaginal Egg Formulations, Devoid of Phages and Integrating Both Phage Particles, by X-ray Fluorescence with Energy Dispersion (EDXRF)

Low and high excitation energies were used to hit the samples, producing emission of elemental characteristic X-rays. The EDXRF spectra of a plain vaginal egg formulation and of a vaginal egg formulation integrating the phage cocktail, at different excitation energies (data not shown), were virtually similar to one another, allowing for the observation that only a slight difference in the spectra was produced at 12 kV excitation energy.

**Figure 13:**
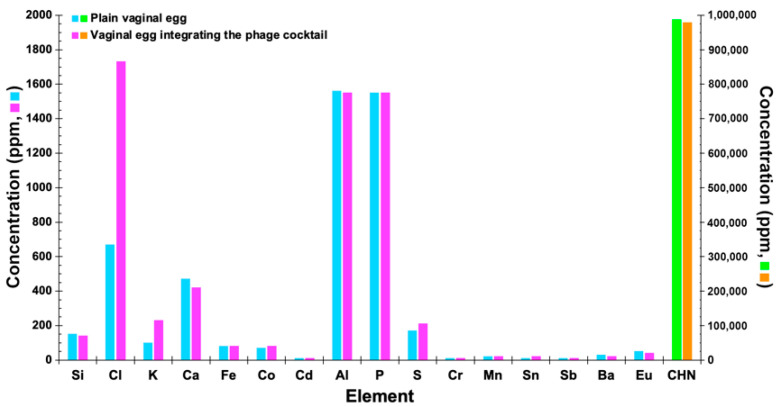
Elemental profile of the plain vaginal egg and of the vaginal egg formulation integrating the phage cocktail, obtained by EDXRF.

The elemental profile of the vaginal egg formulations (Figure 13) presented relatively high concentrations of Cl and K (most likely originating from the bacteriophage suspensions utilized), Al and P (these two elements most likely originating from the cocoa butter itself, with Al being probably a contaminant).

### 3.15. Thermal Characterization of the Vaginal Egg Formulations, Devoid of Phages and Integrating Both Phage Particles, via Differential Scanning Calorimetry (DSC)

**Figure 14:**
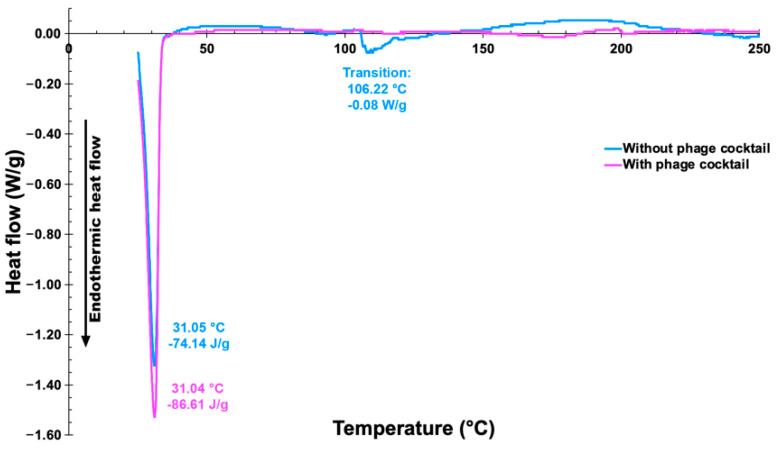
Differential scanning calorimetry thermograms of the plain vaginal egg and of the vaginal egg formulation integrating the phage cocktail.

The DSC thermograms of samples of the vaginal egg formulations, devoid of phages and integrating both phage particles, are illustrated in Figure 14.

A close inspection of the thermograms displayed in Figure 14 allows one to observe very similar thermal events for the two vaginal egg formulations, with the formulation integrating the phage cocktail absorbing a slightly higher amount of energy at exactly the same melting point, viz. 31 °C. The peak temperatures of the two vaginal egg formulations were virtually equal to one another and compare with the mid-point of the melting point range of the cocoa butter matrix (28–35 °C), viz. 31.5 °C.

### 3.16. Mechanical Analyses of the Vaginal Egg Formulations, Devoid of Phages and Integrating Both Phage Particles

**Figure 15:**
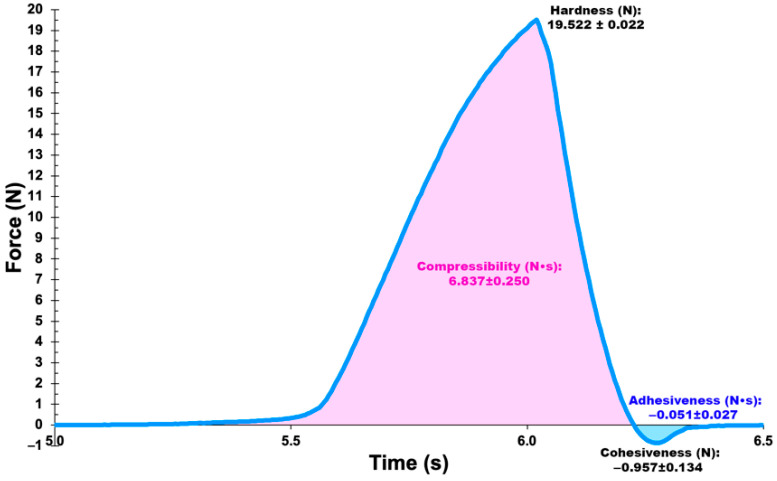
Force-time curve of texture profile analysis together with the results obtained for the mechanical properties of the vaginal egg formulation integrating the cocktail of lytic bacteriophage particles, viz. compressibility, hardness, adhesiveness and cohesiveness.

Evaluation of the mechanical properties of the vaginal egg formulation integrating the phage cocktail encompassed hardness, compressibility, adhesiveness and cohesiveness, parameters obtained from the force-time curve of texture profile analysis (Figure 15), at 20 °C (just below room temperature to avoid melting of the formulation during the analysis).

The compressibility, hardness, adhesiveness and cohesiveness values of the vaginal egg formulation integrating the cocktail of lytic phages, calculated from the force-time data, are displayed in the appropriate locations in the force-time curve (Figure 15). While hardness and cohesiveness were obtained from the ordinate axis in the force-time curve (Figure 15), compressibility and adhesiveness were obtained from the area under the curve, above and below the ordinate axis in the force-time curve (Figure 15), respectively. The results obtained for these mechanical parameters allowed us to confirm the suitability of the vaginal egg formulation for the intended application purpose, viz. transvaginal application and immediate melting and release of the phage particles at the entrance of the uterus cervix.

### 3.17. Surface Morphology of the Vaginal Egg Formulation Integrating Both Phage Particles via Scanning Electron Microscopy (SEM) Analyses

**Figure 16:**
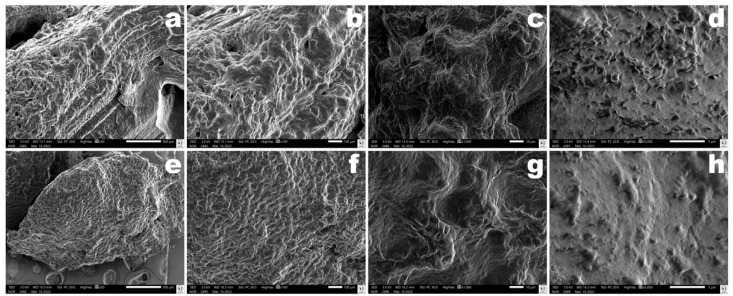
Photomicrographs of the vaginal egg surface at several magnifications ((**a**): ×50, (**b**): ×100, (**c**): ×1000, (**d**): ×5000), and of the cross-section fracture zone ((**e**): ×50, (**f**): ×100, (**g**): ×1000, (**h**): ×5000).

The surface and internal (cryo-fractured zone) morphology of the vaginal egg formulation integrating the cocktail of lytic phage particles was analyzed via scanning electron microscopy, and photomicrographs of its surface and internal morphology are displayed in Figure 16 at different magnifications following lyophilization prior to sputter coating with colloidal gold. The SEM images of both the surface and fractured cross-section of the vaginal egg formulation allows for the observation of a rugged surface with the absence of cracks (Figure 16).

### 3.18. Evaluation of the Cytotoxicity Potential of the Vaginal Eggs Integrating Both Phage Particles, via the Agar Disk-Diffusion Assay

**Figure 17:**
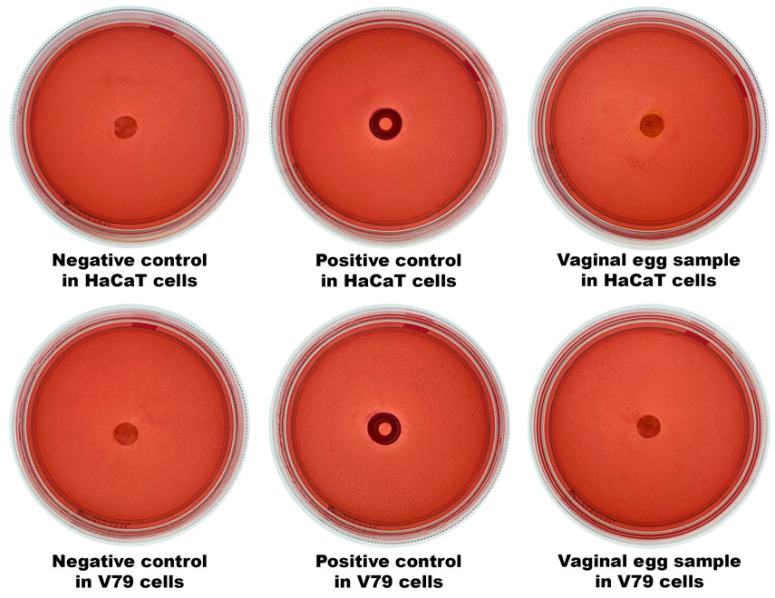
Results obtained in the analysis of the potential for cytotoxicity of the vaginal egg integrating the cocktail of lytic phage particles, via the agar disk-diffusion method with HaCaT and V79 cell lineages.

To carry out these analyses, the cell lines HaCaT (in vitro spontaneously transformed keratinocytes from histologically normal human skin) and V79 (Chinese hamster lung fibroblasts) were used. The results obtained are displayed in Figure 17.

The results gathered in these analyses demonstrated the absence of cell death due to contact, during 24 h, with the vaginal egg sample containing the cocktail of phage virions, with the cells maintaining their integrity following microscope analyses. A clear halo indicative of cell death was only observed for the positive controls, with the cells exhibiting changes in their morphology (Figure 17).

### 3.19. Evaluation of the Storage Stability of the Vaginal Eggs Integrating Both Phage Particles, in Terms of Evolution of Lytic Bioactivity

**Figure 18:**
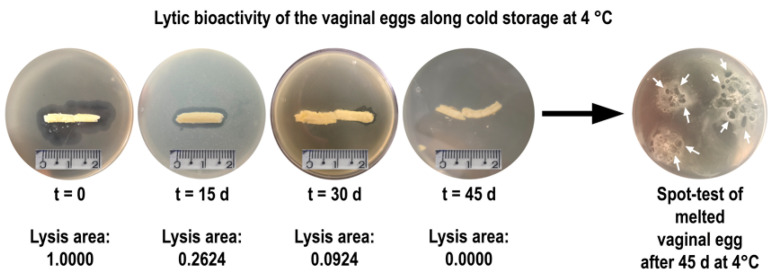
Results from the cold storage stability assessment of the vaginal eggs integrating both phage particles, in terms of evolution of lytic bioactivity. Full scale bar represents 2 cm.

The vaginal egg was maintained under refrigeration for 45 d, aiming at observing the evolution of lytic bioactivity. The results obtained are displayed in Figure 18.

As can be observed from inspection of Figure 18, a clear reduction in the lysis zone was perceived along cold storage, most likely due to an increased dryness of the vaginal egg, preventing the bacteriophage particles from diffusing out of the vaginal egg and contacting with the bacterial host cells in the lawn. However, the dryness of the vaginal egg did not negatively impact upon the lytic activity of the bacteriophage particles, as could be confirmed upon melting the vaginal egg and performing a spot test on a bacterial lawn of the host (Figure 18).

**Figure 19:**
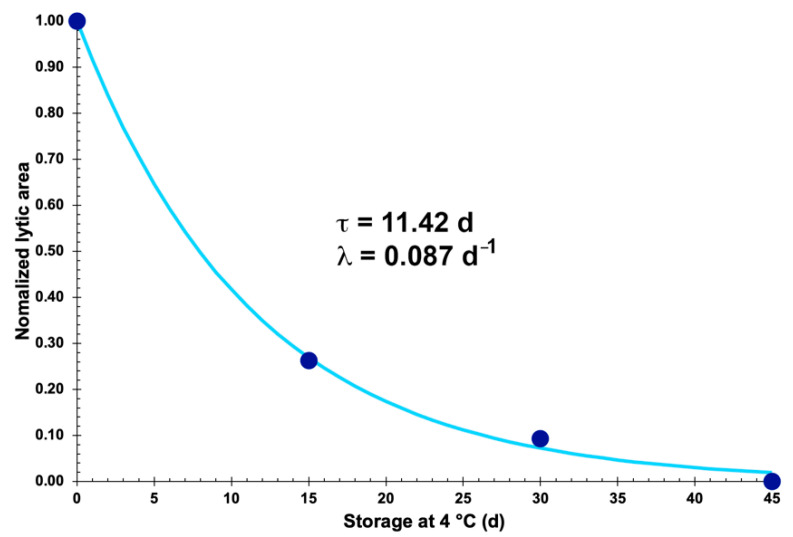
Results from the non-linear fitting of an exponential decay model ($A_{\left(t\right)}/A_{\left(o\right)}=e^{-\left(t/\tau \right)}$) to the evolution of normalized lytic area promoted by the vaginal eggs along cold storage at 4 °C.

From inspection of the non-linear fitting of the exponential decay model performed to the normalized lytic area promoted by the vaginal eggs along cold storage at 4 °C (Figure 19), important parameters such as the decay rate (λ) and the mean lifetime (t = 1/λ) could be determined as 8.7% d^−1^ and 11.42 d, respectively. Despite such decay in the lytic area promoted by the vaginal eggs along cold storage, most likely due to an increased dryness of the formulation, the phage particles integrated into its fat matrix proved to retain their lytic activity, as can be clearly observed in the spot test performed to the melted vaginal egg after 45 d of storage at 4 °C (inserted white arrows in Figure 18).

## 4. Discussion

Developing new alternatives to the conventional antibiotic therapy for preventing and controlling infections by (multidrug-resistant) *E. coli*, has been challenging and a long term goal within the scientific community. In the present study, the structural and functional stabilization of two newly isolated lytic phages for *E. coli* (viz. vB_EcoM_Uniso11 and vB_EcoM_Uniso21, isolated from samples of sewage water from the Veterinary Hospital at UNISO in Sorocaba/SP, Brazil) in a vaginal egg formulation has been proposed, aiming at intrauterine bacterial control in veterinary applications. The results obtained in the present study provide evidence that the use of these phages can reduce the population of pathogenic *E. coli* bacterial cells. The two newly isolated phages produced translucent and dimensionally different plaques on a lawn of the bacterial host, exhibiting diameters between ≈0.5 and ≈1 mm (Figure 2), were identified as members of the order Caudovirales and family *Myoviridae* (Figure 5), displayed distinct molar extinction coefficients (Figure 4b) yet with equal orders of magnitude, and revealed different profiles of structural proteins (Figure 4). The phage plaques were clear and did not exhibit a secondary halo in the frontier of the lysis plaque of phage (Figure 2), which is a likely indication that these phages do not produce depolymerase enzymes. Additional characterization by genome sequencing demonstrated that phages ph0011 and ph0021 were confirmed as different genus members of the *Myoviridae* family (phages with contractile tails) (Table 5). Both phages thus belong to the order Caudovirales and present myovirus-like morphotypes, with phage ph0011 being classified as *Myoviridae*, genus *Asteriusvirus*, and phage ph0021 being classified as *Myoviridae*, genus *Tequatrovirus*, based on their complete genome sequences. Moreover, phages ph0011 and ph0021 genomes do not encode toxins, virulence factors or genes for antibiotic resistance, mandatory features for applications in antibacterial phage therapy [46,99]. The taxonomic lineage of phage vB_EcoM_Uniso11 was confirmed as Viruses → Duplodnaviria → Heunggongvirae → Uroviricota → Caudoviricetes → Caudovirales → Myoviridae → Asteriusvirus, whilst that of phage vB_EcoM_Uniso21 was confirmed as Viruses → Duplodnaviria → Heunggongvirae → Uroviricota → Caudoviricetes → Caudovirales → Myoviridae → Tevenvirinae → Tequatrovirus [81].

The genomes of phages ph0011 and ph0021 do not appear to encode sequences related to depolymerases and, as aforementioned, the lysis plaques they produce (Figure 2) do not exhibit the typical secondary halos surrounding translucent lysis zones that are observed in the case of phages that produce depolymerase enzymes [100].

Moreover, following whole phage genome sequencing and annotation [101], the two phage genomes were screened for the presence of sequences coding integrase enzymes and they did not feature any coding sequences related to lysogeny (integrases), therefore one can safely assume a lytic lifestyle for both phages.

In this work, the host range of the two newly isolated phages was evaluated by determining if they were able to form clear plaques of lysis on particular bacterial strains (hence allowing for the conclusion that if the phages were able to productively infect the bacteria and yield progeny). According to Hyman [101], in addition to the bacterial strain or species used in the isolation procedure, (newly) isolated phage particles may be also able to infect different bacterial cells displaying similar receptors on their surface.

Phages vB_EcoM_Uniso11 and vB_EcoM_Uniso21 were able to bind to most of the *E. coli* bacterial strains from clinical isolates (Table 4) and kill the bacteria, producing a relatively large amount of progeny virions and resulting in the not so low values obtained for the EOP. This was the case for strains from clinical isolates *A1, *A4, *A5, *A7, *A8, *A9, *A11, *A14, *A17 and *A19, representing 53% of the clinical isolates. We speculate, therefore, a phenomenon of lysis from within (with bacterial lysis being induced intracellularly by phage-derived enzymes, such as holins and lysins) as a plausible rationale for the two phages killing these *E. coli* strain cells, which might exhibit some surface receptors that were recognized by the isolated phages. The absence of lysis observed for a few *E. coli* strains from clinical isolates (Table 4, *A6, *A10, *A16, *A22, *A24, *A25 and *A27) might be due either to the expression of phage receptors with different densities and in different locations on the surface of the bacterial outer membrane, hence affecting the phage recognition process, or to bacterial resistance mechanisms against phage infection namely CRISPR [102], a mechanism used by the bacterial host cell to evade infection by phages.

In addition, testing the efficacy of the two newly isolated phages on several other bacterial genera failed to produce positive spots (Table 4). The host range of the two phages seems therefore to be quite narrow, allowing to conclude that natural, non-pathogenic bacteria, will not be affected by antimicrobial treatment with these phage particles. In spite of this, in the future, new phages need to be isolated in order to produce a cocktail with a broad spectrum of activity to control a large number of strains of *E. coli*.

A close Inspection of the phage growth data displayed in Figure 6 allows us to clearly observe a one step of growth for both phages. A good growth can be observed between 20 and 40 min (phage ph0011) or 40 and 50 min (phage ph0021) (Figure 6), levelling off afterwards. Each one of the phage growth curves displayed in Figure 6 represents the average of three independent determinations, producing quite low (asymmetric) standard deviations. The growth features of the two newly isolated phages (Figure 6) exhibited relatively low burst sizes, viz. 106 PFU/host cell (phage ph0011) and 10 PFU/host cell (phage ph0021), indicating that both phages replicate well in *E. coli* CCCD-E003 with short latency periods, viz. 40 min (phage ph0011) and 48 min (phage ph0021). Several studies reported in the literature demonstrated that the use of phages with high burst sizes and short lytic cycles increase the efficiency of phage therapy [103,104,105] but, usually, high burst sizes are accompanied by more extensive latency periods [106]. Phage vB_EcoM_Uniso11 presented the highest burst size, around 10 times higher than that of phage vB_EcoM_Uniso21, and the in vitro phage inactivation of planktonic *E. coli* CCCD-E003 cells was higher with phage vB_EcoM_Uniso11 at a MOI of 1000 (maximum inactivation of 4.01 and 4.24 log CFU/mL for phage vB_EcoM_Uniso11, and 1.90 and 1.90 log CFU/mL for phage vB_EcoM_Uniso21, after 10 h and 12 h of treatment, respectively, Figure 9a1). When both phages were mixed together in a cocktail, bacterial inactivation was most efficient at a MOI of 10 (maximum inactivation of 5.05 log CFU/mL, Figure 9b1), after 12 h of treatment, and at a MOI of 100 (maximum inactivation of 5.57 log CFU/mL, Figure 9b1) after 12 h of treatment. After this timeframe, however, the bacteria acquired resistance to the phage particles at all MOI values tested.

Knowing the dynamics of phage particle amplification inside its bacterial host cell in in vitro experiments is of utmost importance if phage particles are to be used to inactivate pathogenic bacteria. Infection of a susceptible bacterial host cell by a phage particle starts with the virion anchoring onto suitable receptors displayed on the bacterial cell surface [36,107,108,109,110], mostly through a blend of Brownian motion-induced random encounters between phage virions and bacterial host cells, and alterations in the three-dimensional architecture of phage-recognized protein receptors on the host cell surface [35,36,56,111]. Phages ph0011 and ph0021 showed highly similar adsorption rates onto their bacterial host cells, viz. 1.904 × 10^−10^ PFU^−1^ CFU^−1^ mL^−1^ h^−1^ and 1.926 × 10^−10^ PFU^−1^ CFU^−1^ mL^−1^ h^−1^, respectively (Figure 7). These results are one order of magnitude larger than results reported by Moldovan et al. [110] (*E. coli*, l-phage, 1.60 × 10^−11^ cm^3^ s^−1^), one order of magnitude lower than results reported by Heller and Braun [112] (*E. coli*, T5-phage, 1.20 × 10^−9^ mL min^−1^), Zemb et al. [113] (*E. coli*, T2-phage, 1.75 × 10^−9^ mL min^−1^) and Nabergoj et al. [114] (*E. coli* K-12, T4-phage, 2.60 × 10^−9^ mL min^−1^), and of the same order of magnitude of results published by Tsukada et al. [115] (*E. coli*, Qb-phage, 4.00 × 10^−10^ mL min^−1^ cell^−1^) and Høyland-Kroghsbo et al. [116] (*E. coli* l-phage, 2.70–4.90 × 10^−10^ mL min^−1^ phage^−1^ cell^−1^). However, the (first order) phage desorption rate from reversible phage-bacteria complexes was higher for phage vB_EcoM_Uniso21 (1.070 × 10^−2^ mL min^−1^) than for phage vB_EcoM_Uniso11 (5.664 × 10^−3^ mL min^−1^), meaning that less phage vB_EcoM_Uniso21 virion particles remained attached to their host cells. Since attachment of phage particles onto specific surface receptors on the surface of susceptible host cells is mandatory for an efficient infection and subsequent virion morphogenesis [117], the higher desorption rate for phage vB_EcoM_Uniso21 may be in line with the much lower virion morphogenesis yield for this phage (viz. 10 PFU/host cell, Figure 6). The adsorption profile showed that after 30 min, more than 30% and 25% of phages vB_EcoM_Uniso11 and vB_EcoM_Uniso21 particles, respectively, were adsorbed onto the host cells (Figure 7), whereas after 150 min more than 90% and 75% of the phages vB_EcoM_Uniso11 and vB_EcoM_Uniso21 particles, respectively, were adsorbed onto the host cells. As a consequence, phage vB_EcoM_Uniso11 led to a significant decrease in bacterial concentration at MOI 1000, comparing with the non-treated BC, but its effect occurs only after the first 4 h of phage-bacteria incubation (Figure 9a1). During the first 12 h of incubation in the presence of the phage cocktail integrating both phage virions at MOI 100, the bacterial concentration was significantly lower than that observed for the control (Figure 9b1).

To unveil a putative mechanism responsible for the weaker adsorption of phage vB_EcoM_Uniso21 onto the host cells (Figure 7), the Zeta potential and the diffusion coefficient of the phage virions were determined via DLS (Table 3). In Gram-negative bacteria, the outer membrane is surface-decorated with biopolymers containing carboxylic acid and phosphate ester moieties (viz. phospholipids, lipoproteins, lipopolysaccharides and proteins) that are the reason why these bacterial cells display an overall negative charge at neutral pH [113,118]. As can be observed in the data displayed in Table 3, *E. coli* CCCD-E003 cells (in liquid TSB, pH 7.2) showed a large (negative) Zeta potential (viz. −38.49 mV), in clear agreement with results reported elsewhere [113,118] (viz. −60–−10 mV) for pH values between 2.2 and 11, due to carboxylate moieties present in the B-band lipopolysaccharides [118].

Phage particles in suspension might be stabilized due to electrostatic forces, protecting the phages from inactivation due to “charge shielding” [119]. A weaker electrostatic repulsion between *E. coli* CCCD-E003 cells and phage vB_EcoM_Uniso11 (both of which are negatively “charged”), due to a less negative Zeta potential value of the phage vB_EcoM_Uniso11 particles (−8.31 mV, Table 3), might be in line with the results obtained for the higher adsorption rate of this phage onto the bacterial host cells and concomitant higher reduction in bacterial cell numbers (Figure 9a1). A more negative Zeta potential for phage vB_EcoM_Uniso21 (−16.13 mV, Table 3) when compared with the Zeta potential for phage vB_EcoM_Uniso11 (−8.31 mV, Table 3) led to a higher repulsion between its particles and the bacterial cells, resulting in a lower adsorption rate and concomitant lower reduction in bacterial cell numbers (Figure 9a1). Consequently, less negative Zeta potential values for the phage particles produce more favorable interactions between phage particles and planktonic bacterial host cells, intensifying the anti-microbial or killing effect [36,119]. Both newly isolated phages exhibited a negative Zeta potential value, but not as negative as *E. coli* cells, further suggesting that, in addition to the biochemical interactions between phage particles and bacterial cell surface receptors, there is in fact an electrostatic repulsion that hinders an effective contact between both species [36]. *E. coli* cells are generally hydrophobic [120,121] and, depending on the hydrophobicity of the environment, phage particles will also be hydrophobic (because they are constituted mainly by proteins) [119], which suggests that bacterial cells and phage particles will likely repel themselves to different extensions, a result in clear agreement with findings by Harada et al. [36].

As can be observed in Figure 5, the isolated phage particles are not spherical, and therefore the results obtained from the DLS determinations correspond probably to the whole dimension of the phage particles (i.e., their total length, including capsid and tail), which clearly agree with the TEM photomicrographs obtained (Figure 5), since no aggregation effects can be anticipated [113,119] due to the negative Zeta potential values (Table 3).

The two newly isolated phage virions, ph0011 and ph0021, exhibited diffusion coefficients (Table 3) that are highly comparable and of the same order of magnitude (10^−12^ m^2^.s^−1^) as results published elsewhere [36,49,122,123] for both phages and nanoparticles.

Several studies reported in the literature demonstrated that either the reduction in pathogenic bacteria numbers is more pronounced with increasing MOI values, or reduction in bacterial cell numbers occurs quicker at a higher MOI [36,124,125,126]. In the study reported herein, the increase in MOI from 0.1 to 1000 did promote a significant increase in the efficiency of phage treatment, for both phages, after a period of 12 h (Figure 9a1). The initial doses of the phages were not mandatory because of their self-perpetuating feature, disclosed by an increase in phage titers together with bacteria (Figure 9a2). During the 24 h-timeframe of phage-bacteria incubation, the phage virion concentration increased more (by 3.13 log PFU/mL (phage vB_EcoM_Uniso11) and 3.40 log PFU/mL (phage vB_EcoM_Uniso21)) at MOI 100 than at MOI 1 (by 2.63 PFU/mL (phage vB_EcoM_Uniso11) and 1.17 PFU/mL (phage vB_EcoM_Uniso21)) (Figure 9a2). Yet, for the two-phage cocktail during the 24 h-timeframe of incubation with the bacterial host, phage concentration increased more (by 5.19 log PFU/mL) at MOI 10 than at MOI 100 (by 2.21 log PFU/mL) or MOI 1000 (by 2.09 PFU/mL) (Figure 9b2).

*E. coli* was effectively inactivated by the cocktail of both isolated phages (Figure 9b1), reaching the maximum of inactivation of 5.05 log CFU/mL after 12 h of incubation at MOI 10, but unlike phage vB_EcoM_Uniso11, the phage cocktail did not prevent bacterial regrowth (Figure 9b1). At the three MOI tested, a significant bacterial regrowth was observed after 12 h of incubation with the phage cocktail (Figure 9b1). According to several studies reported in the literature, due to the tremendous variation in bacterial surface phage receptors, bacterial regrowth following phage treatment can be surpassed via utilization of a cocktail integrating different lytic phages [50,51,103,127,128,129,130] displaying distinct adsorption mechanisms.

One of the challenges faced when performing bacterial biocontrol studies using phage particles lies in demonstrating its feasibility in real-world matrices [51], and therefore ex vivo phage trials were performed with canine uteri artificially contaminated with *E. coli*. The results gathered in the ex vivo assays (Figure 10a) showed that the phage cocktail integrating the two newly isolated phages inactivated the *E. coli* strain, but the efficacy was lower than that observed in in vitro assays, namely for MOI 10 (2.65 and 4.90 log CFU/mL, respectively, for ex vivo and in vitro after a period of 6 h) and MOI 100 (4.23 and 3.08 log CFU/mL, respectively, for ex vivo and in vitro after a period of 6 h), and for MOI 10 (0.57 and 5.05 log CFU/mL, respectively, for ex vivo and in vitro after a period of 12 h) and MOI 100 (0.89 and 5.57 log CFU/mL, respectively, for ex vivo and in vitro after a period of 12 h), when compared to the nontreated samples.

The ANOVA showed that the differences between sampling time along treatment of *E. coli* with the phage cocktail (*p* < 0.05) and the differences between density of *E. coli* along treatment (*p* < 0.05) were significant when comparing the two MOI (viz. 10 and 100). The phages in the phage cocktail infected and killed *E. coli* cells in canine uterus artificially contaminated. The maximum of *E. coli* inactivation was 1.75 and 3.60 log CFU/mL, for MOI 10 and 100, respectively (Figure 10a, Bonferroni, *p* < 0.05), after 2 h of incubation, whereas the maximum of *E. coli* inactivation was 2.65 and 4.23 log CFU/mL, for MOI 10 and 100, respectively (Figure 10a, Bonferroni, *p* < 0.05), after 6 h of incubation. After 8 h of incubation, the *E. coli* counts (reduction of 2.20 and 2.35 log CFU/mL, for MOI 10 and 100, respectively) were statistically similar (Bonferroni, *p* > 0.05) when compared to the values obtained in the bacterial control. After 12 h of incubation, the *E. coli* counts (reduction of 0.57 and 0.89 log CFU/mL, for MOI 10 and 100, respectively) were also statistically similar (Bonferroni, *p* > 0.05) when compared to the values obtained in the bacterial control (Figure 10a). After the first 8 h of treatment, a significant bacterial regrowth was observed until the end of the incubation timeframe (ANOVA, *p* < 0.05) (Figure 10a) for both MOI values studied. Although phage controls endured fairly constant throughout the phage-bacteria inactivation experiments (ANOVA, *p* > 0.05), when the phage cocktail was incubated with planktonic *E. coli* cells a statistically significant increase in phage concentration (0.53 and 0.72 log PFU/mL, Bonferroni, *p* < 0.05) was observed for MOI 10 and 100, respectively (Figure 10b).

The efficiency of phage therapy depends, to a great extent, on the contact of the phage particles with the bacterial host cells and successful infection and, consequently, on their amplification inside the host [117], leading to cell burst and release of virion progeny. In the ex vivo experiments, the target bacterial cells may be embedded within (or adhered to) the complex matrix of the uterus wall, with concomitant shielding from the phage particles, impairing phage infection and amplification. In fact, as mentioned above, the concentration of phage particles produced in the presence of *E. coli* cells in the in vitro tests (5.19 and 2.21 log PFU/mL, respectively, for MOI 10 and 100 of the cocktail) was higher than that observed in the ex vivo tests (0.53 and 0.72 log PFU/mL, respectively, for MOI 10 and 100 of the cocktail). Hence, when compared to the liquid culture medium in the in vitro assays, the limited diffusion in the uterus solid matrix in the ex vivo assays may have exerted a negative impact on the random contact between bacterial cells and phage particles, putatively preventing phage amplification to a greater extent. Such hindrance may, in part, be circumvented through the use of a larger MOI, implying more phage particles, to perform the treatment. Despite this, there is a major concern with the use of phages to control pathogenic bacteria, due to the eventual emergence of bacterial mutants resistant to the phages and consequent regrowth of bacteria after treatment [51,131]. In this study, for both MOI values tested the bacteria presented mild regrowth between 6 and 12 h of treatment (Figure 10a).

The reduction in *E. coli* concentration by the cocktail integrating both newly isolated phages in the experiments performed in vitro and ex vivo is a fundamental step forward for the deployment of an effective alternative to the antibiotics currently used to treat pyometra. Nevertheless, implementation of this antibacterial strategy in veterinary settings requires more studies using whole uterus, firstly in an in vitro laboratory approach and, afterwards, in vivo with naturally contaminated uterus.

Integration of the phage particles within the cocoa-butter matrix formulation did not interfere with the lytic activity of the entrapped phage particles (Figure 12), a conclusion supported by the clear lysis zones produced by the vaginal egg on the *E. coli* CCCD-E003 bacterial lawn (Figure 12c). This indicated that the immobilized bacteriophage particles, responsible for the lysis phenomena observed, were viable and retained their lytic activity, a conclusion fully supported by previous results from our research group [47,49,132,133]. Despite the fact that the microbiological assays have revealed maintenance of the lytic viability of the immobilized bacteriophage particles, the microarchitecture of the cocoa-butter based matrix with a high degree of compactness will not affect negatively the mobility of the bacteriophage particles towards their bacterial host cells, by providing an almost instantaneous melting of the fatty matrix and release of the phage particles in situ.

Cocoa-fat is a plant-engineered biomaterial which, associated to a small weight percentage of other components so as to produce the vaginal egg, has been found to possess higher elemental contents (K, Cl, Al, P). Al and P were detected in relatively high amounts (Figure 13) and their presence in the vaginal egg sample was most likely due to contaminants originating during the cocoa butter production process. Regarding the differences in Cl content between plain and bioactive vaginal eggs, they were related to the Cl content present in the bacteriophage suspensions utilized to prepare the formulations. Other elements were found in trace amounts in both plain and bioactive vaginal eggs, viz. Fe, Co, Cd, Cr, Mn, Sn, Sb, Ba and Eu, but their origin is most likely related to contaminants present in the deodorized commercial cocoa fat employed to prepare the vaginal egg formulations. As expected, the vaginal egg formulations were composed almost entirely by carbon, hydrogen and nitrogen (Figure 13), arising from the cocoa butter and also from the protein entities integrated in the antibacterial vaginal egg formulation.

The vaginal egg formulations produced are basically made of cocoa butter (>98%, *w*/*w*). Thus, one can observe very similar thermal events for the two vaginal egg formulations, with melting enthalpies of the same order of magnitude, with the formulation integrating the phage cocktail absorbing a slightly higher amount of energy at exactly the same melting point (Figure 14), viz. 31.04 °C. As in most lipids, the main transition observed at 106.22 °C (Figure 14) was from a gel to a liquid crystalline phase [134,135], a fast and highly reversible transition which is characterized by the cooperative melting of the cocoa butter hydrocarbon chains. The energy absorbed during this transition (viz. 0.08 W/g) does not characterize a melting enthalpy, so the transition is most likely second-order. As can be observed from inspection of the plots in Figure 14, the effect on the DSC thermogram curve was only slight and could be observed due to the high sensitivity of the analytical equipment utilized. The major endothermic event in the vaginal egg formulations produced without phage particles and with the cocktail of lytic phage particles is the sharp peaks at 31.05 °C (with associated melting enthalpy of 74.14 J/g) and 31.04 °C (with associated melting enthalpy of 86.61 J/g), respectively (Figure 14). Integration of the phage cocktail in the vaginal egg formulation promoted a slight increase in the melting enthalpy, with the vaginal egg devoid of phage particles and the vaginal egg integrating the cocktail of lytic phage particles displaying peak temperatures virtually equal to one another within a very narrow range, and not so far away from the melting point range of the cocoa butter matrix (28–35 °C) [136,137].

The compressibility, hardness, adhesiveness and cohesiveness of the vaginal egg formulation produced by integrating the cocktail of lytic phages, calculated from the force-time curve of texture profile analysis displayed in Figure 15, are displayed in the appropriate locations in the force-time curve (Figure 15). The results obtained for these mechanical parameters allowed to confirm the suitability of the formulation for the intended purpose. The hardness of the vaginal eggs formulated in the research work entertained herein was ca. 19.5 N, a value smaller than the range obtained by Kishino et al. [138,139] for vaginal suppositories, viz. 26–43 N. The low value obtained for the (muco)adhesiveness of the vaginal egg endowed with antibacterial properties will exert a large influence on the permanence of the lytic phage particles in the intended application site, since the vaginal egg is intended for transvaginal application and immediate melting and release of the phage particles, without adhering to the entrance of the uterus cervix (but without entering the cervix), results opposite to those intended for human vaginal suppositories carrying chemical drugs [140,141]. The low value obtained for the compressibility of the vaginal egg was in line with the semi-solid nature of the formulation since, due to the fact that a small amount of aqueous solvent (vehiculated with the phage suspensions) was added, a water-in-oil emulsion was produced which integrated the formulation. Regarding the low cohesiveness produced (Figure 15), in line with the low value for the (muco)adhesiveness, it is vital for the virtually instant melting of the vaginal egg at the entrance of the uterus cervix. Hence, the results obtained in terms of (not so high) hardness, low compressibility and low (muco)adhesiveness and low cohesiveness are all in line with the intended application for vaginal eggs endowed with antibacterial activity. While “in-the-packaging” features such as low(er) compressibility and high(er) hardness, together with low adhesiveness and low cohesiveness are desired at room temperature, the same features are intended for “out-of-the-packaging” at the intended temperature of use, viz. ca. 37 °C.

In addition, from the results of the microstructural and morphological analysis gathered in the scanning electron microscopy analyses performed to the vaginal egg formulation, a uniform surface morphology could be observed (photomicrographs in Figure 16a–d). From the observation of the fractured cross-section of the cocoa butter matrix (photomicrographs in Figure 16e–h), a highly uniform and compact matrix structure can be clearly seen. These observations are very significant, since the phage particles were uniformly dispersed within the cocoa butter matrix, and the photomicrographs in Figure 16 allow for the clear observation of the compactness of the formulation, without air pockets that could negatively impact phage viability.

No cell death caused by contact with the vaginal egg sample containing the cocktail of bacteriophage particles could be observed (Figure 17).

The results described herein clearly suggest that antibacterial treatment utilizing the two newly isolated lytic phages, ph0011 and ph0021, has the potential to be an effective surrogate to antibiotics in controlling *E. coli.* Yet, unlike phage ph0011, phage ph0021 could not fully refrain bacterial regrowth. Hence, selecting lytic phages for phage-bacteria inactivation assays should consider not only their efficacy but also the potential for development of phage-resistant bacterial mutants. Testing the in vitro phage-bacteria inactivation effectiveness assumes therefore a particular relevance prior to moving on to in vivo phage-bacteria inactivation assays. In addition to this, integrating different lytic phages for the same bacteria in a cocktail with concomitant supplementation of the narrow host-range characteristics of the two newly isolated phages would allow one to circumvent and virtually obliterate the downside of bacterial-acquired resistance to phages.

## Figures and Tables

**Table 1:** Host range of the two newly isolated phages determined on 41 bacterial strains. Clear lysis zone (+) and no-lysis zone (−). An EOP value of 100% was considered for the host strain.

Bacterial Strains	Source	Phage vB_EcoM_Uniso11		Phage vB_EcoM_Uniso21	
		Spot Test	EOP (%)	Spot Test	EOP (%)
*E. coli* CCCD—E003	Collection, CEFAR	+	100 (host)	+	100 (host)
*Salmonella enterica* CCCD—S004	Collection, CEFAR	-	-	-	-
*Pseudomonas aeruginosa* CCCD—P004	Collection, CEFAR	-	-	-	-
*Proteus mirabilis* CCCD—P001	Collection, CEFAR	-	-	-	-
*Enterococus faecalis* CCCD—E002	Collection, CEFAR	-	-	-	-
*Bacillus subtilis* CCCD—B010	Collection, CEFAR	-	-	-	-
*Staphylococcus epidermis* CCCD—S010	Collection, CEFAR	-	-	-	-
*Staphylococcus aureus* CCCD—S009	Collection, CEFAR	-	-	-	-
*Klebsiella pneumoniae* CCCD—K001	Collection, CEFAR	-	-	-	-
*E. coli* ATCC-25922	Collection, ATCC	+	44.33	+	37.66
*Enterobacter* sp. (*2.2)	Collection, Labiton-UNISO	-	-	-	-
*Enterobacter aerogenes* (*2.13)	Collection, Labiton-UNISO	-	-	-	-
*Klebsiella pneumoniae* (*4.15)	Collection, Labiton-UNISO	-	-	-	-
Staphylococcus intermedius*	Collection, PhageLab-UNISO	-	-	-	-
*Pseudomonas syringae* pv. syringae*	Collection, PhageLab-UNISO	-	-	-	-
*Pseudomonas syringae* pv. garcae*	Collection, PhageLab-UNISO	-	-	-	-
*Xanthomonas axonopodis* pv. *citri* 306	Collection, PhageLab-UNISO	-	-	-	-
*Proteus penneri* (*5.5)	Collection, Labiton-UNISO	-	-	-	-
*Proteus vulgaris* (*5.4)	Collection, Labiton-UNISO	-	-	-	-
*E. coli* (*3.2)	Collection, Labiton-UNISO	+	12.89	+	9.54
*E. coli* (*3.4)	Collection, Labiton-UNISO	+	45.66	+	41.34
*E. coli* (*3.5)	Collection, Labiton-UNISO	-	-	-	-
*E. coli* (*A1)	Clinical isolate, non-castrated female cat	+	55.66	+	59.36
*E. coli* (*A4)	Clinical isolate, non-castrated female cat	+	48.21	+	39.18
*E. coli* (*A5)	Clinical isolate, female dog with pyometra	+	10.89	+	12.18
*E. coli* (*A6)	Clinical isolate, female dog with pyometra	-	-	-	-
*E. coli* (*A7)	Clinical isolate, female dog with pyometra	+	26.81	+	32.64
*E. coli* (*A8)	Clinical isolate, female dog with pyometra	+	70.66	+	71.12
*E. coli* (*A9)	Clinical isolate, female dog with pyometra	+	29.33	+	19.54
*E. coli* (*A10)	Clinical isolate, female dog with pyometra	-	-	-	-
*E. coli* (*A11)	Clinical isolate, female dog with pyometra	+	45.34	+	52.33
*E. coli* (*A14)	Clinical isolate, female dog with pyometra	+	88.23	+	76.54
*E. coli* (*A16)	Clinical isolate, female dog with pyometra	-	-	-	-
*E. coli* (*A17)	Clinical isolate, non-castrated female cat	+	59.66	+	44.18
*E. coli* (*A19)	Clinical isolate, non-castrated female cat	+	64.54	+	70.12
*E. coli* (*A21, #1)	Clinical isolate, mare	+	0.00016	+	0.0000005
*E. coli* (*A22, #2)	Clinical isolate, mare	-	-	-	-
*E. coli* (*A24, #4)	Clinical isolate, mare	-	-	-	-
*E. coli* (*A25, #5)	Clinical isolate, mare	-	-	-	-
*E. coli* (*A26, #6)	Clinical isolate, mare	+	0.00014	+	0.000002
*E. coli* (*A27, #7)	Clinical isolate, mare	-	-	-	-

**Table 2:** Composition of the formulation utilized to prepare the vaginal eggs integrating a cocktail of phages vB_EcoM_Uniso11 and vB_EcoM_Uniso21 particles.

Components	Function in the Formulation	Amount (mg)		% (*w*/*w*)
		Plain Vaginal Eggs	Antibacterial Vaginal Eggs	
Cocoa butter	Lipid matrix	9842	9842	98.42
Concentrated phage VB_EcoM_US_11 suspension (2.38 × 10^11^ PFU/mL)	Active antibacterial entities	0	9 (9 µL, containing 2.142 × 10^9^ virions)	0.180
Concentrated phage VB_EcoM_US_21 suspension (2.12 × 10^11^ PFU/mL)		0	9 (9 µL, containing 1.908 × 10^9^ virions)	
Ultrapure water	Plasticizer	18	0	
Tween 80	Nonionic (polysorbate) surfactant to help stabilize the bacteriophage particles	40	40	0.400
Preservative (methylparaben, 6 g; propylparaben, 3 g; propylenglycol, 91 g)	Preservative, antifungal	100	100	1.000
TOTAL:		10,000	10,000	100.00

**Table 3:** Results from the bacterial identification via biochemical tests performed to the colonies isolated from canine uterine samples with pyometra, and from mares.

Clinical Sample	Origin	Gram Coloration	Lactose Fermentation	Oxidase	Citrate	Gas	Indol	Lysin	Motility	H_2_S Production	Urea	Identification
A1	Cat	-	+	-	-	+	+	+	-	-	-	*E. coli*
A2	Canine	+	n.p.	n.p.	n.p.	n.p.	n.p.	n.p.	n.p.	n.p.	n.p.	*Streptococcus* sp.
A3	Canine	-	+	-	+	+	-	+	-	-	+	*Klebsiella pneumoniae*
A4	Cat	-	+	-	-	+	+	-	-	-	-	*E. coli*
A5	Canine	-	+	-	-	+	+	+	+	-	-	*E. coli*
A6	Canine	-	+	-	-	+	+	+	+	-	-	*E. coli*
A7	Canine	-	+	-	-	+	+	+	-	-	-	*E. coli*
A8	Canine	-	+	-	-	+	+	+	+	-	-	*E. coli*
A9	Canine	-	+	-	-	+	+	+	-	-	-	*E. coli*
A10	Canine	-	+	-	-	+	+	+	-	-	-	*E. coli*
A11	Canine	-	+	-	-	+	+	+	+	-	-	*E. coli*
A12	Canine	-	-	-	-	-	-	-	-	-	-	No growth
A13	Canine	-	-	-	+	-	-	-	+	+	+	*Proteus mirabilis*
A14	Canine	-	+	-	-	+	+	-	+	-	-	*E. coli*
A15	Canine	-	+	-	+	+	-	+	-	-	+	*Klebsiella pneumoniae*
A16	Canine	-	+	-	-	+	+	+	+	-	-	*E. coli*
A17	Cat	-	+	-	-	+	+	-	+	-	-	*E. coli*
A18	Canine	-	+	-	+	+	-	+	-	-	+	*Klebsiella pneumoniae*
A19	Cat	-	+	-	-	+	+	+	+	-	-	*E. coli*
A20	Canine	-	+	-	+	+	-	+	-	-	+	*Klebsiella pneumoniae*
A21 (#1)	Mare	-	+	-	-	+	+	+	-	-	-	*E. coli*
A22 (#2)	Mare	-	+	-	-	+	+	+	-	-	-	*E. coli*
A23 (#3)	Mare	-	-	-	+	+	-	+	+	-	+	*Serratia marcescens*
A24 (#4)	Mare	-	+	-	-	+	+	+	-	-	-	*E. coli*
A25 (#5)	Mare	-	+	-	-	+	+	+	-	-	-	*E. coli*
A26 (#6)	Mare	-	+	-	-	+	+	-	+	-	-	*E. coli*
A27 (#7)	Mare	-	+	-	-	+	+	-	+	-	-	*E. coli*

Note: n.p.—not performed

**Table 4:** Results obtained for the physical parameters of phages vB_EcoM_Uniso11 and vB_EcoM_Uniso21.

Physical Parameter	Phage Particles		*E. coli* CCCD-E003 Cells
	vB_EcoM_Uniso11	vB_EcoM_Uniso21	
Hydrodynamic size (nm)	310.76 ± 25.40	109.09 ± 3.68	607.52 ± 4.92
Polydispersity Index	0.383 ± 0.027	0.384 ± 0.023	0.349 ± 0.024
Zeta Potential (mV)	−8.31 ± 1.12	−16.13 ± 2.64	−38.49 ± 0.32
Electrophoretic Mobility (µ/s)/(V/cm)	−0.650 ± 0.085	−1.260 ± 0.212	−3.010 ± 0.030
Diffusion Coefficient (m^2^ s^−1^)	7.39 × 10^−12^ ± 6.27 × 10^−14^	2.25 × 10^−12^ ± 7.45 × 10^−14^	4.04 × 10^−13^ ± 3.26 × 10^−15^

**Table 5:** Genomic features of phages ph0011 and ph0021 genomes.

Feature	Phage vB_EcoM_Uniso11 (Phage ph0011)	Phage vB_EcoM_Uniso21 (Phage ph0021)
NCBI/Genbank accession number	OP557969	OP557970
Genome size	348,288 bp	165,222 bp
Number of PE reads	23,449,832	20,127,644
Number of PE reads mapping in the final assembly	11,724,916 (50.0%)	10,063,822 (50.0%)
Average sequencing coverage	33.7×	60.9×
GC content	45%	43%
tRNA genes	7 + 1 pseudogene	11 + 3 pseudogenes
Protein-coding genes (CDS) predicted	575	264
With function assigned	128 (22.3%)	245 (92.8%)
Hypothetical/unknown function	447 (77.7%)	19 (7.2%)
Closest phage genome sequence	*E. coli* phage vB_EcoM_phAPEC6	*E. coli* phage vB_EcoM_WL-3
NCBI accession number of similar phage	MK817115.1	MT968995.1
Family/Genus	*Myoviridae/Asteriusvirus*	*Myoviridae/Tequatrovirus*

## Data Availability

The phage genome sequences described in this work have been deposited in GenBank NCBI (National Center for Biotechnology Information) under accession numbers OP557969 (ph0011) and OP557970 (ph0021).

## Supplementary Materials

The following supporting information can be downloaded at: https://www.mdpi.com/article/10.3390/pharmaceutics14112344/s1, Table S1. UV-Vis_spectrophotometric_data_phages_ph0011_ph0021; Table S2. annotated_genomes_phages_ph0011_ph0021.
