# Supplementary material for: Isolation and Molecular Characterization of Two Novel Lytic Bacteriophages for the Biocontrol of Escherichia coli in Uterine Infections: In Vitro and Ex Vivo Preliminary Studies in Veterinary Medicine
Source: Pharmaceutics. 2022 Oct 30;14(11):2344. doi: 10.3390/pharmaceutics14112344 (PMC9692438; doi:10.3390/pharmaceutics14112344)
Supplement: Supplementary file 1 [file pharmaceutics-14-02344-s001.zip › Supplementary_Table_S1_UV-Vis_spectrophotometric_data_phages_ph0011_ph0021.pdf]

Supplementary Table S1. Data utilized to prepare calibration curves aiming at determining the molar extinction coefficients of phages vB_EcoM_Uniso11 and vB_EcoM_Uniso21 (whole) particles.

Concentrated phage suspension (μL)	Final volume of dilution (μL)	Phage	Number of PFU's in the concentrated phage suspension	Phage particle concentration (PFU/mL)	$Abs_{255\ nm}$	$Abs_{320\ nm}$	Abs_{255nm}-Abs_{320nm}
25	2000	ph0011	$4.9750x10^{08}$	$2.4875x10^{08}$	0.5988	0.2121	0.3867
		ph0021	$6.1000x10^{08}$	$3.0500x10^{08}$	0.5697	0.0784	0.4913
50	2000	ph0011	$9.9500x10^{08}$	$4.9750x10^{08}$	0.7499	0.3032	0.4467
		ph0021	$1.2200x10^{09}$	$6.1000x10^{08}$	0.8732	0.2534	0.6198
100	2000	ph0011	$1.9900x10^{09}$	$9.9500x10^{08}$	1.1568	0.5236	0.6332
		ph0021	$2.4400x10^{09}$	$1.2200x10^{09}$	1.1254	0.3992	0.7262
150	2000	ph0011	$2.9850x10^{09}$	$1.4925x10^{09}$	1.4330	0.6839	0.7491
		ph0021	$3.6600x10^{09}$	$1.8300x10^{09}$	1.5369	0.6286	0.9083
200	2000	ph0011	$3.9800x10^{09}$	$1.9900x10^{09}$	1.8420	0.9364	0.9056
		ph0021	$4.8800x10^{09}$	$2.4400x10^{09}$	1.9838	0.8754	1.1084
